# Analysis of classical techniques precision on the measurement of cellulose moisture gain/loss

**DOI:** 10.3389/fchem.2023.1254941

**Published:** 2023-09-06

**Authors:** Stefan Cichosz, Anna Masek, Katarzyna Dems-Rudnicka

**Affiliations:** ^1^ Faculty of Chemistry, Institute of Polymer and Dye Technology, Lodz University of Technology, Lodz, Poland; ^2^ Centre of Mathematics and Physics, Lodz University of Technology, Lodz, Poland

**Keywords:** cellulose, water content, infrared spectroscopy, thermogravimetric analysis, Karl-Fischer titration, boxplot

## Abstract

The precision of the four classical techniques (Karl-Fischer titration, (thermo)gravimetric method, Fourier-transform infrared (FT-IR) and near infrared (NIR) spectroscopies) commonly used in the analysis of cellulose moisture absorption/desorption has been deeply investigated regarding the reproducibility of these processes. Based on multiple repeated experiments, cellulose water content values obtained with Karl-Fischer titration and (thermo)gravimetric method were plotted as a function of time. Then, the cautious peak-by-peak analysis of the absorbance and wavenumber shifts visible in IR spectra has been carried out. The collected data was described using boxplots that provided valuable information on the experimental points spread. It has been successfully proven that gravimetric methods allow for precise drawing of moisture absorption and desorption curves, while Karl-Fischer titration, ATR FT-IR and NIR techniques provide the possibility of the moisture absorption/desorption processes description by linear mathematical models (R2 >90%). Therefore, this study provides a systematic comparison between various analytical methods.

## 1 Introduction

The following study has been the attempt to assess the utility of some analytical techniques in the investigation of barely reproducible cellulose moisture absorption/desorption processes. Cellulose, as a biopolymer of plant origin, is characterized by high heterogeneity in structure and properties (Bledzki et al., 1996). Depending on the source of origin (e.g., a specific plant species), as well as obtaining from different parts of the plant, cellulose may differ, e.g., in the length of macromolecules, the degree of crystallinity or the specific surface area (Thygesen et al., 2005; Gašparovič et al., 2010; Huber et al., 2012). In turn, these structural changes affect the properties of the biopolymer (Ibrahim et al., 2013; Chirayil et al., 2014; Matykiewicz et al., 2021). Therefore, the processes of moisture absorption/desorption by cellulose fibres are heterogeneous and their repeatability is relatively low (Lin, 1991; Khoshtinat et al., 2021). Consequently, the rate of water absorption can vary at different stages of the moisture gain/loss process (due to saturation of the material), between the samples (effect of structural diversity), and also at different stages of the process between the samples (synergistic effect).

Different measurement methods may show varied sensitivity to the changes described above. For this reason, it is highly important to determine the precision of the measurements used in relation to the heterogeneity of the analysed processes of moisture absorption/desorption by cellulose fibres. Basing on the literature research presented below, Karl-Fischer titration, gravimetric analysis and IR spectroscopy have been chosen for further investigation regarding their availability, applicability and utility in moisture content determination of cellulose-based materials, as well as the possibility of providing some additional scientific information. Below a literature review has been presented.

### 1.1 Gravimetric methods

Gravimetric analysis describes a set of methods used in analytical chemistry for the quantitative determination of an analyte based on its mass. The principle of this type of analysis is that once an analyte mass has been determined as a unique compound, that known measurement can then be used to determine the same analyte’s mass in a mixture, as long as the relative quantities of the other constituents are known.

To begin with, Osunkoya et al. (2007) merely derived wood water content from the ratio of the difference between wet and dry mass to the wet mass and expressed as a percentage. Similarly,Seifert et al. (2004) also employed gravimetrical methods in their work deliberating on the possibilities of controlling the water content of never dried and reswollen bacterial cellulose. Nevertheless, the measurement procedure has been extended due to the specificity of the tested material. Samples were stirred in distilled water for 2 h at 30°C, centrifugated (m_wet_), and dried at 105°C until a constant weight (m_dry_) was obtained. Then, the values of the water content were calculated according to the following equation: (m_wet_ - m_dry_)/m_wet_^.^ 100.

Moreover, it was proven that gravimetric methods might be favourably applied not only while measuring the water content at one point in time, but also when the continuous changes in the water content are being analysed. Chaurasia et al. (2010) investigated water sorption properties of zinc oxide nanoparticles-loaded cellulose acetate films via recording the weight differences as a function of time. Pre-weighted films were placed in Petri dishes inside glass desiccators containing different saturated salt solutions, thus providing a constant relative humidity environment ranging from 3%–98%. The desiccators were placed inside a temperature-controlled incubator. Then, the samples were weighed at different time intervals using an electronic balance.

### 1.2 Karl-Fischer titration

Karl-Fischer titration is a classic titration method in chemical analysis that uses coulometric or volumetric titration to determine trace amounts of water in a sample. It was invented in 1935 by the German chemist Karl Fischer. It might be used in coulometric and volumetric modes. The main compartment of the coulometric titration cell contains the anode solution and the analyte. In this type of titration, the anode solution consists of an alcohol $\left(ROH\right)$ , a base $\left(B\right)$ , ${SO}_{2}$ and KI . Additionally, the titration cell also consists of a smaller compartment with a cathode immersed in the anode solution of the main compartment. Importantly, the two compartments are separated by an ion-permeable membrane. The reactions taking place are shown in Eqs 1–3. Firstly, the anode generates $I_{2}$ from the KI when current is provided through the electric circuit. Then, an oxidation of ${SO}_{2}$ by $I_{2}$ occurs. One mole of $I_{2}$ is consumed for each mole of $H_{2}O$ . In other words, 2 mol of electrons are consumed per mole of water. The end point is detected most commonly by a bipotentiometric titration method.

$$2I^{-}\to I_{2}+2e^{-}$$ (1)

$$B∙I_{2}+B∙{SO}_{2}+B+H_{2}O\to 2BH^{+}I^{-}+{BSO}_{3}$$ (2)

$${BSO}_{3}+ROH\to BHRSO_{4}$$ (3)

Similarly, the volumetric titration is based on the same principles as the coulometric titration, except that the anode solution above now is used as the titrant solution. Therefore, the titrant consists of an alcohol $\left(ROH\right)$ , base $\left(B\right)$ , ${SO}_{2}$ and a known concentration of $I_{2}$ . Then, 1 mol of $I_{2}$ is consumed for each mole of $H_{2}O$ . Finally, the end point may be detected by a bipotentiometric method.

Karl-Fischer titration has been used by Mazza et al. (2009) who investigated the influence of water on the dissolution of cellulose in selected ionic liquids. The solutions of cellulose in ionic liquids were prepared at elevated temperatures to evacuate the ambient water. Next, the solutions were put into vials with a magnetic stirrer and water was added discretely into the vials. The water content in each vial has been established with Karl-Fischer titration.

### 1.3 IR spectroscopy methods

Generally speaking, infrared spectroscopy (IR spectroscopy or vibrational spectroscopy) is the measurement of the interaction of infrared radiation with matter by absorption, emission, or reflection. The use of this technique in the quantitative analysis of water content in cellulose samples is not obvious. However, it was exploited by Hou et al. (2014) in analysis of water content in cellulose-based material. The researchers have investigated the kinetics of water diffusion through the ethyl cellulose (EC) films plasticized with triethyl citrate (TEC). The films analysed were casted from alcoholic solutions of EC/TEC, dried and then subjected to the water absorption experiment. The spectral data has been collected as a function of time with acquisition interval of 40 s. Next, the normalized ATR FT-IR spectra of dried EC-based films containing from 0–30 wt.% TEC were investigated. The authors focused mainly on the region 3,700–3,100 cm ^-1^ that is closely related to the O-H vibration of water molecules (Oh et al., 2005a). It has been observed that the intensity of the O-H stretching band was gradually increasing in the first 2 h for all the investigated diffusion systems. Then, the researchers calculated the integrated areas of the O-H stretching region and plotted it as a function of diffusion time in order to obtain water diffusion curves. Importantly, on the basis of Fickian model and collected data, the authors succeeded in determination of the water diffusion coefficients for different systems analysed.

Similarly, Consumi et al. (2021) have come to congruous conclusions. The researchers investigated carboxymethyl cellulose (CMC) of a varied water content and found that the intensities of the bands related to the stretching vibration of OH group (broad absorption band at 3,392 cm^−1^ and a shoulder at 3,255 cm^−1^) were significantly affected by the water presence. The authors determined the total area under the ATR FT-IR curve in the range 3,675–2,980 cm^−1^ and plotted it against the water content in cellulose sample. A simple linear regression including all the experimental data and IR band areas showed a good fit. Moreover, a satisfying correlation between the measured and predicted data was found for all the five training sets.

Nonetheless, Célino et al. (2014) have concluded that not only a broad peak from 3,700–3,000 cm^−1^ might be useful in the quantitative analysis of water content in cellulose-based systems. The authors investigated the spectral signatures of various raw plant fibres, namely, hemp, flax and sisal at different water contents. These plant-originated materials are widely known to be good candidates for the reinforcement of polymer-based composite materials (Bledzki and Gassan, 1999). The authors described the molecular effect of water sorption mechanisms and employed a multivariate model linking different regions of FT-IR spectra. Firstly, the researchers performed Kruskal Wallis analysis on each individual wavenumber of the raw spectra. It highlighted several zones of the FT-IR fingerprint that were strongly impacted by the increasing water uptake: i) the broad band situated between 3,600–3,000 cm^−1^; ii) the maximum at 1,635 cm^−1^; iii) the band placed in the wavenumber ranging from 1,100–700 cm^−1^. Surprisingly, according to the Kruskal Wallis *p*-values assignment carried out by the researchers, the CH stretching band situated at 2,935–2,900 cm^−1^ was also found to be significantly impacted by the water uptake. However, the models were calculated promptly from the information contained in the spectral regions situated from 3,600–3,000 cm^−1^ and around 1,650 cm^−1^. According to the authors, these maxima were associated to the main regions characteristic of the water-biopolymer interactions, while the remaining absorption bands have been excluded and assigned to the materials’ intrinsic variability.

Furthermore, Yuan et al. (2019) also tried to determine different IR regions useful in the quantitative analysis of the water content in cellulose-based systems. Nevertheless, the authors of this work have employed a slightly different approach. The researchers presented an interesting study on the water content determination in TEMPO oxidized cellulose nanocrystal film (TOCNF). Water content of TOCNF was collected using the dynamic vapor sorption (DVS) apparatus. Then, the results were correlated with recorded ATR FT-IR spectra. The researchers noted that the main band at 3,347 cm^−1^, assigned to the OH group, has changed significantly during the water absorption process. Meanwhile, the weaker maximum located next to 1,608 cm^−1^ and attributed to C-O-C asymmetric stretching vibration was observed to shift toward lower wavenumbers. Moreover, the authors also pointed out that with an increasing relative humidity the intensity of the band at 1,171 cm^−1^ has been gradually falling down while the absorbance of the peak situated at 1,159 cm^−1^ was rising up. Based on this, the researchers concluded that primarily three spectrum ranges correlate with the water adsorption in cellulose-based materials: 3,700–3,000 cm^−1^, 1,700–1,580 cm^−1^ and 1,180–1,140 cm^−1^.

### 1.4 Infrared thermography (IRT)

In turn, Ludwig et al. (2004) took an advantage of another spectroscopic technique. The scientists investigated the possibilities of water detection in wood and plaster by IR thermography (IRT). Using a thermocamera as a reflectometer, the comparison between the reflectance of the standards and the reference samples permitted the evaluation of the optical absorbance in the tested samples. The performed research allowed to determine the most adequate IRT procedures for detecting the moist areas in masonry and wooden structures.

Nonetheless, Madruga et al. (2020) have gone a one step further and presented an interesting study on the measuring the water content in wood using step-heating thermography and speckle patterns. IRT was able to retrieve thermal parameters of the wood related to the amount of water added to the samples, while the interference pattern generated by speckles was used to quantify the expansion and contraction of wood that can be related to the amount of water. By applying advanced image processing to thermograms and specklegrams, it was possible to determine fundamental values controlling the absorption of water.

### 1.5 A brief summary and thoughts

On the basis of the presented literature review, it can be seen that (thermo)gravimetric techniques, Karl-Fischer titration and IR spectroscopy are the most commonly used. Therefore, these analytical methods have been chosen for the cautious statistic-rooted investigation of their accuracy in description of cellulose moisture absorption/desorption. Although the literature is rich in descriptions of the processes of water gain/loss by cellulose fibres and methods that can be used for this purpose, there is still little information on the legitimacy of their use.

Consequently, this study provides statistically-supported data on the precision of the mentioned analytical techniques in relation to the reproducibility of the cellulose moisture gain/loss. Therefore, water absorption/desorption experiments were repeated twenty-five times and the collected data was carefully described using boxplots and, where possible, regression models. The approach presented has been used for the first time in this kind of study. It allowed to favourably determine the application potential of the studied laboratory techniques in quantitative analysis, as well as indicated the repeatability of the moisture absorption/desorption processes and the accuracy of the methods used. Therefore, the information gathered undoubtedly provides a scientific novelty.

## 2 Materials and methods

### 2.1 Materials

Cellulose fibres of a length between 6–12 μm, trade name Arbocel UFC100 Ultrafine Cellulose for Paper and Board Coating, with a density referred as 1.3 g/cm^3^ was delivered by J. Rettenmaier & Söhne (Rosenberg, Germany). Its specific surface area has been determined as approx. 4 m^2^/g with a total pore volume of about 0.02 cm^2^/g and WAXS-calculated crystallinity at the level of 60%. Moreover, phosphorus oxide (V) and potassium nitrate were purchased from Chempur (Poland, Piekary Slaskie) and employed as the desiccator cartridges for, respectively, cellulose conditioning and moisture absorption experiment. Phosphorous oxide (V) is a solid that exhibits pH of approximately 1.5 and density of about 2.3 g/cm^3^. It is referred to provide an atmosphere of the relative humidity (RH) of approximately 0% (Mihranyan et al., 2004b). In turn, the solubility of potassium nitrate in water is on the level of 316 g/L (20°C) and, according to literature, its saturated aqueous solution is able to provide the atmosphere of a specific relative humidity which is approximately 96% (Mihranyan et al., 2004b). Additionally, Hydranal Solvent E and Hydranal Titrant 5E, used during Karl-Fischer titration experiment, were supplied by Honeywell Fluka (Loughborough, UK).

### 2.2 Preparation of cellulose specimens of a varied moisture content

Cellulose studied in this research has been divided between separate weighing bottles (35 × 70 mm) with 0.1 g (Fourier-transform infrared spectroscopy, near infrared spectroscopy, thermogravimetric analysis) or 1.5 g (Karl Fischer titration, weight changes detection) of cellulose in each vessel. Importantly, a separate weighing bottle for a separate experiment has been prepared. Next, cellulose conditioning, moisture absorption and desorption experiments were carried out: i) cellulose conditioning in the desiccator filled with phosphorus oxide (RH = 0%) for 7 days (all prepared weighing bottles with cellulose); ii) moisture absorption experiment: desiccator filled with saturated solution of potassium nitrate; experiment lasted 24 h, measurements after: 0, 1, 3, 5, 8, 24 h (the last measurement after 24 h is the beginning of the moisture desorption stage at 0 h); iii) moisture desorption experiment: laboratory dryer (Binder, Tuttlingen, Germany) at 100°C for 8 h, measurements after: 0, 1, 3, 5, 8, 24 h. Time intervals between the measurements have been selected on the basis of previously reported data (Cichosz and Masek, 2020a). In order to obtain reliable results, the described above processes of cellulose moisture absorption/desorption were repeated multiple times. Therefore, the results presented are the values averaged from 25 experimental points.

### 2.3 Determination of cellulose properties

#### 2.3.1 Karl-Fischer titration

Karl-Fischer titration was done with the use of TitroLine Alpha (Schott, Mainz, Germany) device. For each experiment approximately 1.5 g of cellulose sample and 30 mL of Hydranal Solvent E were taken.

#### 2.3.2 Detection of weight changes

During the carried out measurements, cellulose samples of an initial weigh of approximately 1.5 g were tested. In both absorption and desorption experiments, the analytical moisture analyser Radwag MA 50/1.R.WH supplied by Merazet (Poznan, Poland) have been employed. Specimens’ mass has been recorded for 8 h with the 15 min interval. As mentioned above, m oisture absorption experiment was performed with the use of desiccator filled with saturated solution of potassium nitrate. The cellulose specimen has been taken out each 15 min in order to determine its weight. Then, the stage of moisture desorption was carried out with the use of moisture analyser set at 100°C (the moisture analyser is equipped with a heating source). Sample’s mass was read each 15 min. Then, normalized cellulose mass ( NCM ) has been established according to Eq. 4 and Eq. 5, respectively, for water desorption and absorption analysis:

$$NCM=\left\{\begin{array}{c} \frac{m_{t}-m_{e}}{m_{0}-m_{e}}\;\left(4\right)\\ \frac{m_{t}-m_{0}}{m_{e}-m_{0}}\;\left(5\right) \end{array}\right.$$

where: $m_{t}$ – specimen mass at the time *t* [g] $m_{e}$ – sample mass at the end of experiment [g] $m_{0}$ – initial sample mass [g]

#### 2.3.3 Thermogravimetric analysis

Thermogravimetric analysis (TGA) has been performed in the temperature range from 25°C–600°C (heating rate: 10°C/min; Ar 60 cm^3^/min). Mettler Toledo TGA/DSC 1 STARe System equipped with Gas Controller GC10 has been employed in this investigation. Samples were placed in the zinc oxide crucibles. Additionally, values of moisture desorption activation energy ( $E_{A}$ ) were calculated according to Eqs 6–10 with the use of Broido’s method (Broido, 1969):

$$y=\frac{m_{t}-m_{\infty }}{m_{0}-m_{\infty }}$$ (6)

$$\ln\left[\ln\left(\frac{1}{y}\right)\right]=-\frac{E_{A}}{R}∙\frac{1}{T}+C$$ (7)

as a linear function:

Y=aX+b (8)

where:

$$Y=\ln\left[\ln\left(\frac{1}{y}\right)\right],X=\frac{1}{T},a=-\frac{E_{A}}{R},b=C$$ (9)

therefore

$$E_{A}=-a∙R$$ (10)

where: $m_{t}$ – specimen mass at the time *t* [g] $m_{0}$ – specimen mass at the beginning of considered decomposition step [g] $m_{\infty }$ – specimen mass at the end of considered decomposition step [g] T – temperature [K] R – gas constant [8.31 J/(mol^.^K)]

#### 2.3.4 Fourier-transform infrared spectroscopy (FT-IR)

Fourier transform infrared spectroscopy (FT-IR) absorbance spectra were recorded within the 4,000–400 cm^−1^ range. In order to ensure an acceptable signal-to-noise ratio, 64 scans at resolution of 4 cm^−1^ were accumulated (absorption mode). The experiment has been performed with the use of Thermo Scientific Nicolet 6700 FT-IR spectrometer equipped with diamond Smart Orbit iTX attenuated total reflection (ATR) sampling accessory (Waltham, MA, USA). Recorded spectra were baseline corrected with OMNIC 9.2.86 software.

#### 2.3.5 Near infrared spectroscopy (NIR)

Near infrared spectroscopy (NIR) absorbance spectra were recorded within the 10,000–4,000 cm^−1^ range. In order to ensure an acceptable signal-to-noise ratio, 64 scans at resolution of 4 cm^−1^ were accumulated (absorption mode). The experiment has been performed with the use of Thermo Scientific Nicolet 6700 (Thermo Scientific), equipped with Smart NIR Integrating Sphere Accessory (InGaAs detector). Recorded spectra were baseline corrected with OMNIC 9.2.86 software.

#### 2.3.6 Data analysis

**FIGURE 1:**
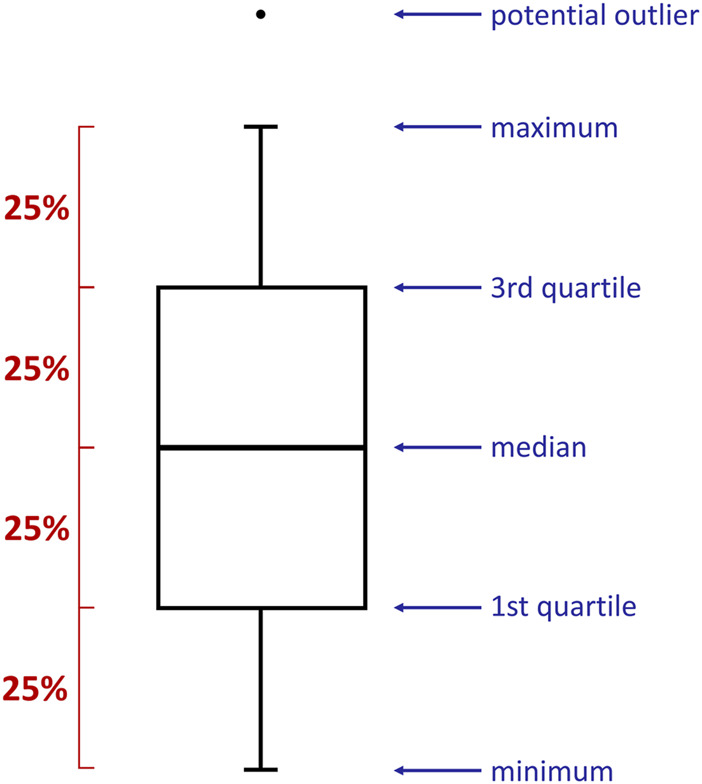
A boxplot is a standardized way of displaying the spread of data by revealing: minimum, first quartile (Q1), median, third quartile (Q3), and maximum. Importantly, each part of the box contains 25% of the data population.

The relationship between the time and some specific parameters have been presented in the form of boxplots (Figure 1). They provided valuable information on the collected data distribution, e.g., minimum/maximum value, median, quartiles. Boxplots were prepared with *RStudio* (version 1.4.1106) software (R Core Team, 2021) using *ggplot2* library [install.packages(“ggplot2”)] and *reshape2* package [install.packages(“reshape2”)]. The data was imported into the program from previously prepared *.csv* files. The following commands have been exploited: data <- data.frame(read.csv2(file)), boxplot(data, names = “”), data_long <- melt(data), ggplot(data_long, aes(variable, value)) + geom_boxplot(), ggplot(data_long_labels, aes(variable, value)) + geom_boxplot(). Importantly, each box presented in this research has been established based on 25 reproduced measurements.

Moreover, if applicable, the adjustment of linear fits was tested regarding the reliable description of the water content-time relationship. Therefore, two models were investigated using least squares method, namely, simple linear (Eq. 11) and semilogarithmic (Eq. 12):

Y=a∙X+b (11)

$$Y=a∙\ln\left(X\right)+b$$ (12)

Y stands for an investigated parameter and X is time. The fits selected have been established on the basis of 5 experimental points, each of which reflects the value averaged from 25 repeated measurements. Firstly, the performance of simple linear model was estimated using the adjusted coefficient of determination (R^2^). If it was impossible to describe a relationship with simple linear model (R^2^ < 90%), the possibility of linearization of the analysed dependence was investigated using the semi-logarithmic function. The applied value logarithmization is a mathematical operation that allows reducing the dependencies that do not show linearity to a linear function. However, it does not exhibit any physical sense (it is a simple mathematical operation).

Again, analysis of the data has been performed with *RStudio* (version 1.4.1106) software (R Core Team, 2021). The data was imported into the program from previously prepared *.csv* files, and then the analysis of individual linear models was performed along with the assessment of their adequacy using the program’s built-in functions. Firstly, the simple linear model was assessed. If the analysed data did not show a linear relationship, semilogarithmic model was examined. Moreover, graphs revealing linear models were prepared using *ggplot2* package [install.packages(“ggplot2”)]. Furthermore, the following commands have been used: simple linear – model <- lm( Y ∼ X , data = data_file), semilogarithmic – model <- lm( Y ∼ log( X ) , data = data_file).

## 3 Results and discussion

### 3.1 Karl-Fischer titration

**FIGURE 2:**
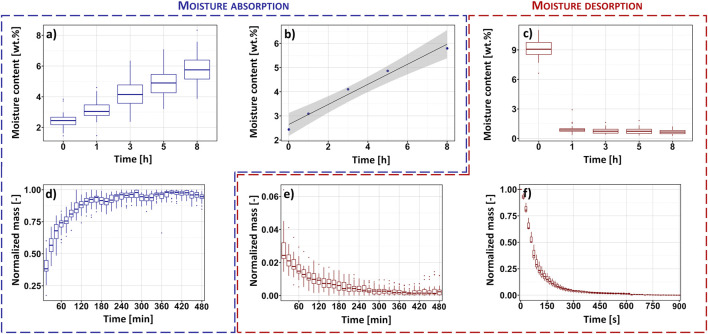
Results of Karl-Fischer titration experiments: averaged moisture content plotted as a function of moisture content during moisture absorption **(A)** and desorption **(C)** processes, as well as the linear relationship between cellulose water amount and moisture absorption time **(B)**. Additional comparison with the recorded sample weight changes in time **(D–F)**.

Firstly, moisture content has been determined with Karl-Fischer titration technique. The data gathered during the analysis performed is presented in Figures 2A–C. Importantly, Figures 2A, C reveal the changes in moisture content over time, while cellulose subjected to, respectively, moisture absorption and desorption processes. Additionally, Figure 2B shows a linear model fitted to the experimental points reflecting the averaged water content plotted as a function of moisture absorption time.

It is easily detectable from Figure 2A that the water content of cellulose elevates in a gradual and steady manner as the time of moisture absorption increases. Moreover, by disclosing the spread of collected data, the boxplot applied reveals the possibly higher reproducibility of moisture content measurements till 1 h of the carried out experiment. Then, from 3 h, the data dispersion notably broadens, hence, indicating different water absorption rates between the repeated experiments and lower precision of this method. Consequently, as the measurements were carried out in the same conditions, it might be assumed that the speed of the absorption processes depends on the sample itself.

This could possibly relate to the nature of plant-originated materials. Being a natural biopolymer, cellulose is characterized by a high degree of structural diversity (Burgert and Fratzl, 2009; Kondo et al., 2016; Zhu et al., 2016; Hospodarova et al., 2018) which might undeniably affect the repetitiveness of the processes analysed. However, another possibility which cannot be neglected is that the error observed origins from the measurement itself (observational error). Thus, underlining the importance of the studies on the reproducibility of the properties of plant-derived substances and the legitimacy of the methods used.

Additionally, moving further towards Figure 2B, a graph with experimental points and a regression line might be observed. Dark blue dots represent the values of moisture content averaged from 25 repetitions and assigned to the specific time moment from the beginning of the moisture absorption experiment. The black line reflects the fitted linear regression and the grey colour symbolises the confidence region. From Figure 2B it might be easily noticed that despite the data variance, a simple linear model has been successfully fitted. Exhibiting a high value of adjusted determination coefficient R^2^ = 97.4%, the function proposed reliably describes the relation between the cellulose water content determined with Karl-Fischer titration and moisture absorption time. This finding indicates the possibility of quantitative assessment, as well as observation of cellulose moisture content in time based on a few experimental points. However, the regression has been examined only for the water content in the range from 0.5–11.0 wt.% and needs to be extensively investigated in the future.

Then, the moisture desorption experiment has been performed in a laboratory dryer at the temperature of 100°C which is a commonly known water boiling point. The results of the carried out analysis have been presented in Figure 2C. It might be easily perceived that cellulose moisture content dramatically decreases during the first hour of an experiment and then remain relatively constant. Importantly, the reproducibility of the carried out measurements seems to be significantly higher in comparison with the previously examined moisture absorption (narrow data spread) experiment. However, it should be also considered that the Karl-Fischer titration measurements were carried out with relatively long time intervals. Therefore, all moisture content variations recorded, especially during first hour of the experiment, are not entirely visible.

### 3.2 Detection of weight changes with time

Fortunately, gravimetric measurements allow to increase the frequency of the readings, and thus enable a more accurate description of the processes of cellulose moisture absorption/desorption. By recording the changes in the mass of the samples, not only the increase or decrease in mass caused by the moisture content changes, but also the repeatability of these processes can be observed.

The results of performed analysis are presented in Figures 2D–F. Similarly to the previous subsection, firstly, moisture absorption experiment has been investigated. Looking carefully at the graph shown in Figure 2D, which illustrates the weight gain during the moisture absorption process, one can observe some differences from the results obtained by the Karl-Fischer titration method (Figure 2A). Previously, the variations in moisture content over time could have been described using a simple linear function (Figure 2B). Yet, the experimental data presented in Figure 2D does not form a straight line. Changes in the moisture content form a curve-like shape that reaches an equilibrium after approx. 300 min of the measurement. Moreover, the boxplot shown in Figure 2D indicates the broad dispersion of recorded experimental points at the beginning of moisture absorption process (wider boxes) and not while the water amount in cellulose is elevated. This observation differs from what has been previously recorded regarding the Karl-Fischer titration-derived results (Figure 2A). These changes could have been caused by the different frequency of measurements and the lower sensitivity of the Karl-Fischer titration method to variations in moisture content compared to the gravimetric method. However, this issue requires further investigation.

Nonetheless, the data gathered during the experiment of moisture desorption by the means of gravimetric and Karl-Fischer titration methods correlates with each other – the shapes created by the experimental points in Figures 2C, E, F are alike and all exhibit broadened spread of results at the beginning of the analysed process. Hence, indicating similar accuracy of these techniques in the moisture content determination during water desorption process.

Figure 2E reflects the changes in the sample’s weight during the whole desorption experiment (8 h). However, the first box (initial measurement) has been skipped to better visualise further mass changes. This choice was dictated by the large differences in sample’s weight at the beginning of the measurement. Yet, the first 15 min (900 s) of the experiment were carefully examined in a separate investigation the results of which are presented in Figure 2F. While comparing the data shown in Figures 2C, E, F describing the same process in a parallel way, some similarities might be found. First of all, the data indicates the high reproducibility of desorption process from the first hour of the measurement (narrow boxes). Moreover, considering both analytical techniques, the experimental points form a hyperbole-like shape. This indicates fast water desorption from cellulose fibres and quickly achieved equilibrium state.

### 3.3 Thermogravimetric analysis (TGA)

Additionally, the cellulose samples subjected to moisture absorption and desorption experiments have been investigated via thermogravimetric analysis (TGA). Unfortunately, this analytical method does not provide the possibility of carrying out measurements at short intervals. The authors are aware of the possibility of using the technique of thermogravimetric analysis (TGA) in isothermal mode. However, such an arrangement would only work during the moisture desorption process. For this reason, for comparative purposes, it was decided to keep the same methodology for the analysis of both moisture gain and loss processes. Therefore, with this highly accurate method the water amount in cellulose fibres have been examined and determined for two types of specimens, namely,: i) after 8 h of moisture absorption experiment (wet), ii) after 8 h of moisture desorption experiment (dry). In this way, not only was the amount of water known after the complete moisture absorption/desorption experiment, but also the reproducibility of these processes was cautiously assessed.

**FIGURE 3:**
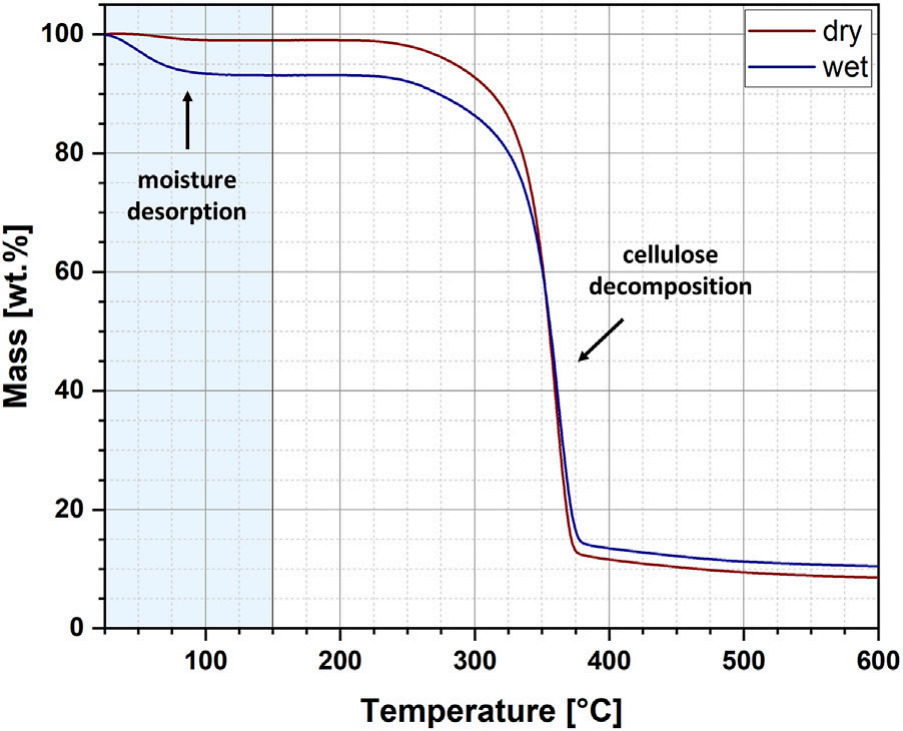
Exemplary thermogravimetric curves for the wet and dry cellulose specimens.

The exemplary thermogravimetric curves recorded for wet and dry cellulose samples have been revealed in Figure 3. The curves show two weight loss steps. The first is related to the desorption of water and is marked with a light blue colour. In turn, the second one has been assigned to the thermal decomposition of cellulose. Importantly, in this research, only the first weight loss step was analysed and further statistical description of obtained results has been presented in Table 1. For the analysed samples, two parameters were determined: moisture content and activation energy of the water desorption process. Moreover, the data has been carefully described by the following statistical values: mean, 95% lower confidence interval (LCI), 95% upper confidence interval (UCI), standard deviation and coefficient of variation.

**TABLE 1:** Results of the thermogravimetric analysis (TGA) reflecting the moisture content in wet and dried cellulose samples.

Sample	Parameter	Mean	95% lower confidence interval (LCI)	95% upper confidence interval (UCI)	Standard deviation [-]	Coefficient of variation [%]
Wet	Moisture content [wt.%]	5.16	4.87	5.45	0.70	13.6
	Moisture desorption activation energy [kJ/mol]	63.85	63.08	64.61	1.86	2.9
Dried	Moisture content [wt.%]	1.92	1.69	2.16	0.57	29.8
	Moisture desorption activation energy [kJ/mol]	72.80	70.76	74.85	4.96	6.8

From the data presented in Figure 3 and Table 1 some interesting conclusions could have been made. To begin with, the moisture content of cellulose samples after the performed water absorption experiment has been established at approximately 5.16% and after the water desorption experiment – about 1.92%. These observations corresponds with the results obtained from Karl-Fischer titration. Moreover, the obtained values of standard deviations (SD) might indicate a higher repeatability of the desorption processes (SD = 0.57) as compared to the absorption processes (SD = 0.70), which also confirms previous observations made with gravimetric measurements.

However, the standard deviation measures only how far the average value lies from the mean. In turn, the coefficient of variation measures the ratio of the standard deviation to the mean. The higher the coefficient of variation, the higher the standard deviation of a sample relative to the mean. This means that, according to data given in Table 1, the moisture absorption processes exhibit a deviation of only about one-tenth of the mean value (coefficient of variation is approx.10%). On the other hand, although the moisture desorption processes seem to be characterized by increased reproducibility (lower value of the standard deviation), the obtained results vary up to one third of the mean value (coefficient of variation at the level of about 30%). This, undeniably, states for the higher reproducibility of the water content measurements during the moisture absorption process.

Additionally, the calculated values of activation energies for the desorption of moisture contained in cellulose fibres indicate a reduction in the energy needed for water evaporation when dealing with a wet sample. The observed variations may be related to different interactions of water molecules with natural fibres which has been extensively described in literature (Weise et al., 1996; Peng et al., 2012).

These differences in intermolecular interactions between cellulose macromolecules and water can lead to variations in the activation energy needed to destroy the resulting hydrogen bonds and desorb the moisture. It is highly probable that cellulose in the dried state contains water molecules closely bound to the surface of the fibre through a specific network of hydrogen bonds formed between the cellulose hydroxyl groups and water. Consequently, more energy must be provided to destroy these interactions and evaporate the moisture. A different situation may arise when the moisture content in natural fibres is increased (after the moisture absorption process has been carried out). Then a large part of the water absorbed by the cellulose is free water, which does not require a large amount of energy to destroy the resulting interactions. Quite simply, these interactions do not exist, and unbound water exhibits the same properties as free water. However, it is only a theoretical assumption and the existence of three types of water in dried and not dried cellulose samples should be deeper investigated in the future.

### 3.4 Fourier-transform infrared (FT-IR) and near infrared (NIR) spectroscopy

**FIGURE 4:**
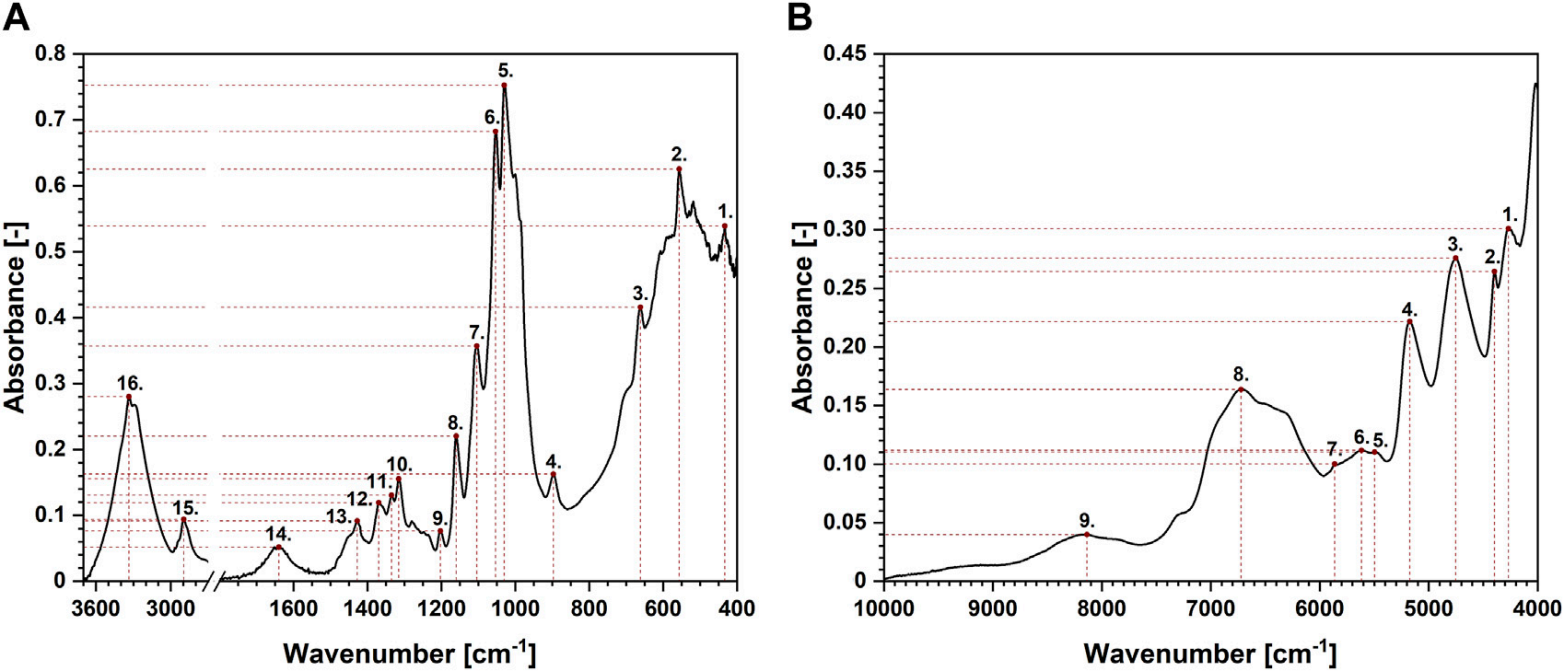
Exemplary attenuated total reflectance Fourier-transform infrared **(A)** and near infrared **(B)** spectra of cellulose with the assignment of the peaks analysed.

Another technique that might be favourably employed in the analysis of moisture absorption/desorption by cellulose-based fibres is infrared spectroscopy. It is characterized by a high sampling rate, simultaneously providing some indispensable scientific information on the chemical moieties embodied in the investigated compound. Absorption bands taken into consideration in this study have been shown in Figures 4A, B. Selected maxima were marked with red dots. Each red dot represents a point regarded for further mathematical analysis and the dashed lines reveal how the coordinates (X – wavenumber, Y – absorbance) have been read. Moreover, Table 2 gathers information about an assignment of chemical moieties present in cellulose fibres to the selected absorption bands.

**TABLE 2:** Assignment of the analysed absorption bands to chemical groups present in the cellulose structure.

Peak no.	Wavenumber range [cm^−1^]	Chemical group	Ref.
Fourier-transform infrared spectroscopy (4,000–400 cm^−1^)			
1.	444–432	vibration of C-O bonds	Fengel, 1998; Kaur et al. (2012)
2.	560–556	γCH, characteristic of cellulose I	Chen et al. (2020)
3.	668–660	δCOH out of plane	Lee et al. (2015)
4.	900–895	γCOC at β-glycosidic linkage; amorphous region	Oh et al. (2005b)
5.	1,032–1,027	γCO at C-6	Lee et al. (2015)
6.	1,056–1,051	γCO at C-6	Lee et al. (2015)
7.	1,107–1,103	γ ring in plane	O’Connell et al. (2010)
8.	1,162–1,159	γCOC at β-glycosidic linkage	Oh et al. (2005b)
9.	1,203–1,199	δCOH in plane at C-6	Oh et al. (2005b)
10.	1,316–1,314	δCH_2_ (wagging) at C-6	Poletto et al. (2014)
11.	1,335–1,332	δCOH in plane at C-2 and C-3	Olsson and Salmén (2004)
12.	1,370–1,360	δCH	Ciolacu et al. (2011)
13.	1,429–1,425	δCH_2_ (symmetric) at C-6; crystalline region	Oh et al. (2005b)
14.	1,646–1,636	absorbed water (hydrogen-bonded)	Oh et al. (2005b)
15.	2,898–2,888	γCH	Morán et al. (2008)
16.	3,339–3,327	γOH covalent bond, hydrogen bonding	Oh et al. (2005a)
Peak no.	Wavenumber range [cm^-1^]	Chemical group	Ref.
Near infrared spectroscopy (10,000–4,000 cm^−1^)			
1.	4,274–4,268	deformation of CH_2_	Caliari et al. (2017)
2.	4,397–4,394	stretching of C-H	Krongtaew et al. (2010), Caliari et al. (2017)
3.	4,758–4,749	stretching of -OH	Krongtaew et al. (2010), Caliari et al. (2017)
4.	5,190–5,170	stretching/bending of -OH, water	Krongtaew et al. (2010), Yan et al. (2013), Caliari et al. (2017)
5.	5,470–5,490	2nd overtone stretching of -OH and C-O	Pinto et al. (2016), Caliari et al. (2017)
6.	5,620–5,600	1st overtone stretching of C-H	Krongtaew et al. (2010), Yan et al. (2013)
7.	5,866–5,855	1st overtone stretching of C-H	Krongtaew et al. (2010), Pinto et al. (2016)
8.	6,740–6,700	1st overtone stretching of -OH	Rodriguez-Saona et al. (2000), Caliari et al. (2017)
9.	8,200–8,110	2nd overtone stretching of C-H and CH_2_	Yan et al. (2013)

In turn, Table 3 shows the results of the adjustment of mathematical models to the changes in absorbance and wavenumber values of the maxima visible in IR spectrum of cellulose fibres. Simple linear fit and semilogarithmic regression were tested. To clearly show the adjustment of mathematical models, values of adjusted determination coefficient (R^2^) have been given. Additionally, the results were divided into two parts reflecting the moisture absorption and desorption processes. Then, the data has been furtherly subdivided considering the absorbance- and wavenumber-based models.

**TABLE 3:** Tabularized values of determination coefficients (R^2^) assigned to the mathematical models based on the results obtained from Fourier-transform infrared (FT-IR) and near infrared (NIR) spectroscopy.

Peak no.	Calibration for moisture absorption				Calibration for moisture desorption			
	Absorbance-based		Wavenumber-based		Absorbance-based		Wavenumber-based	
	Linear model R^2^ [%]	Semilogarithmic model R^2^ [%]	Linear model R^2^ [%]	Semilogarithmic model R^2^ [%]	Linear model R^2^ [%]	Semilogarithmic model R^2^ [%]	Linear model R^2^ [%]	Semilogarithmic model R^2^ [%]
Fourier-transform infrared spectroscopy (FT-IR)								
1.	66.2	96.7	0.1	49.7	14.6	87.5	0.1	48.9
2.	66.6	97.1	62.5	98.8	16.3	88.5	10.0	82.3
3.	66.7	97.2	49.0	99.0	15.5	88.0	16.8	90.3
4.	68.8	96.5	68.7	57.0	13.4	86.3	0.0	65.7
5.	67.2	97.3	28.4	93.1	19.7	90.0	12.7	83.8
6.	68.4	97.0	66.2	93.7	19.8	90.1	18.6	90.0
7.	70.0	96.4	8.5	0.1	18.5	89.2	0.1	0.0
8.	70.9	96.0	0.1	71.4	17.0	88.4	0.1	69.7
9.	74.0	94.4	47.6	97.1	12.6	85.3	17.7	90.7
10.	71.3	95.5	5.0	46.8	15.4	87.7	0.1	0.2
11.	71.4	95.6	60.4	60.2	15.2	87.5	8.1	84.2
12.	70.3	96.0	43.3	99.1	15.3	87.6	7.2	84.6
13.	71.0	95.7	34.7	81.1	14.5	87.2	0.0	0.3
14.	69.1	93.9	14.4	76.4	12.4	86.2	77.6	69.0
15.	66.2	97.7	60.7	94.7	15.2	87.8	28.8	95.2
16.	69.5	95.8	98.9	51.0	15.2	88.0	12.5	53.7
Near infrared spectroscopy (NIR)								
1.	53.3	99.1	46.7	95.8	0.1	65.0	16.9	90.6
2.	54.1	99.1	73.0	82.8	0.3	62.6	16.6	87.6
3.	52.9	99.2	75.1	91.6	0.1	58.4	25.0	89.8
4.	47.5	98.2	0.1	78.6	13.2	87.2	22.4	91.8
5.	60.9	98.0	0.1	0.2	2.5	78.8	0.2	44.4
6.	59.5	98.3	3.4	69.7	3.6	79.8	0.3	45.1
7.	61.4	97.7	63.5	95.7	3.9	79.8	0.1	0.4
8.	57.5	98.2	27.8	95.4	2.4	78.8	11.0	84.2
9.	65.7	70.8	72.8	10.1	0.8	70.8	14.6	89.4

It was assumed that if the determination coefficient is greater than 90%, the mathematical model is characterized by a reliable representation of the experimentally obtained results. Therefore, taking into account the data summarized in Table 3, it might be stated that it is possible to successfully describe the changes in the peak’s position using semilogarithmic models analysed in this study.

Interestingly, maxima visible in FT-IR spectrum originated from both oxygen-embodying (peaks no.: 1, 3, 4, 5, 6, 8, 9, 11, 14, 16) and oxygen-lacking (peaks no.: 2, 7, 10, 12, 13, 15) chemical groups responded mathematically-describable to the variations in moisture content in cellulose fibres. Similar situation has been denoted for NIR spectroscopy: signals assigned to both oxygen-rich (peaks no.: 3, 4, 5, 8) and carbon-based (peaks no.: 1, 2, 6, 7, 9) chemical moieties were influenced by the variations in cellulose water content. Further ATR FT-IR and NIR spectra interpretation led to the conclusions alike. Therefore, for legibility reasons, only the changes in ATR FT-IR spectrum have been discussed in the main part of the article and the remaining data might be found in Supplementary Material.

It is not surprising that changes in the maxima assigned to oxygen-rich chemical moieties, being able to form hydrogen bonds with water molecules, altered during the water absorption process (Hofstetter et al., 2006; Salmén and Bergström, 2009). However, it is not certain what kind of a mechanism stands behind the recorded variations in absorbance/wavenumber of the peaks assigned to non-polar CH_2_ and C-H chemical groups that are not able to directly interact with water molecules. Nevertheless, Celino et al. (Célino et al., 2014) proposed a theory that could bring some elements of understanding. The changes observed might be favourably described with the surrounding signals originated from oxygen-embodying chemical moieties. The shoulders of the maxima assigned to carbon- and oxygen-rich chemical groups overlap, hence, affecting the shape, as well as the height of the peaks visible in IR spectrum (a synergistic effect).

**FIGURE 5:**
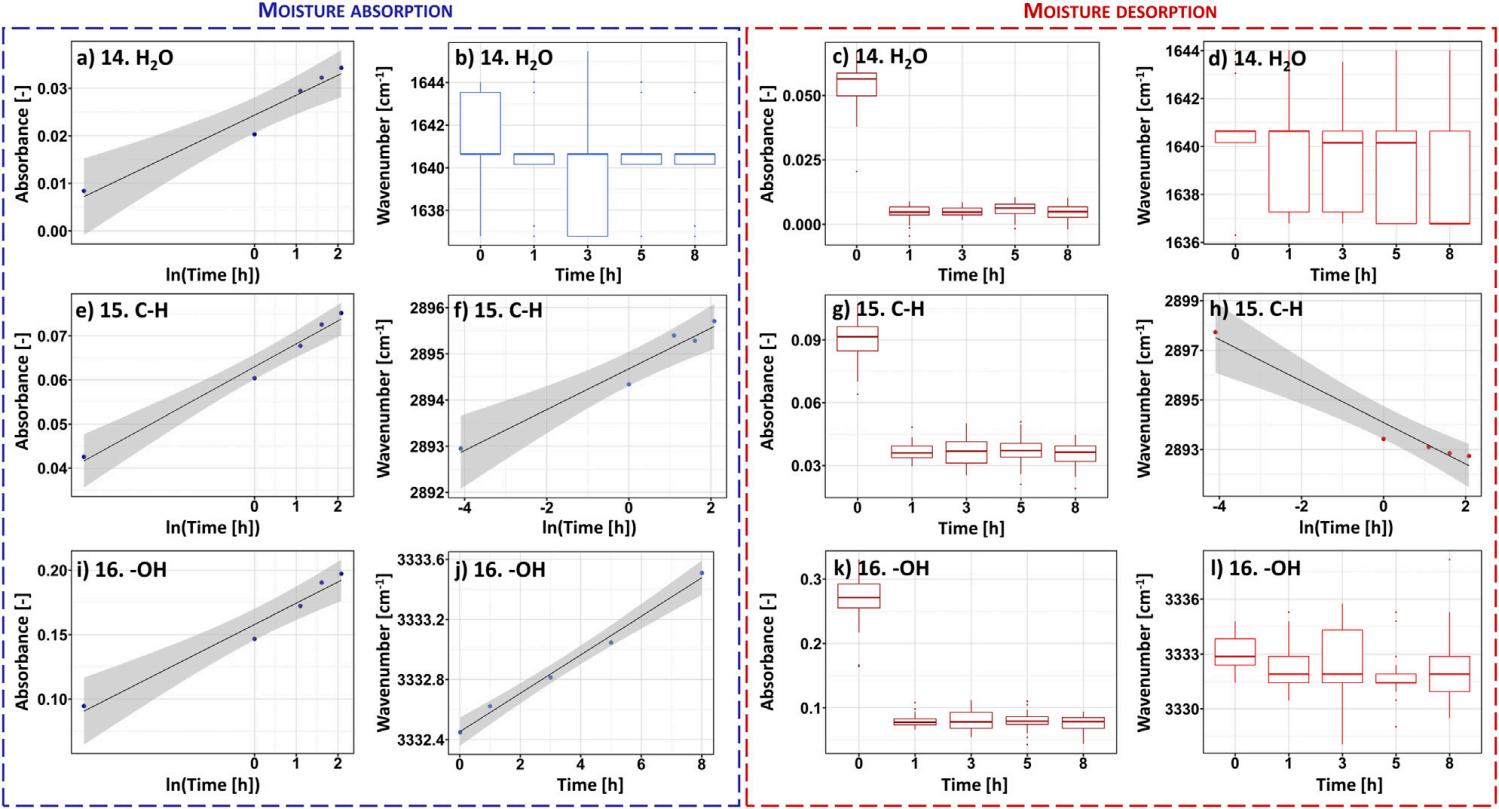
Selected results of the analysis carried out for the ATR FT-IR recorded data revealing the shifts in absorbance/wavenumber values during the processes of moisture absorption and desorption: peak no. 14 assigned to the presence of water molecules, peak no. 15 attributed to C-H groups, peak no. 16 reflecting the vibrations of hydroxyl moieties. Full data available in *Supplementary file*.

Moving forward, to better visualize the possibility of fitting mathematical models to chemical groups with and without oxygen atoms, Figure 5 has been prepared. It shows the data assigned to the FT-IR-derived peaks no.: 14, 15 and 16 which are attributed to, respectively, water molecules presence (Oh et al., 2005b), C-H moieties (Morán et al., 2008) and hydroxyl chemical groups (Oh et al., 2005a).

Regarding Figure 5 regressions were revealed where it was possible to apply a linear model (R^2^ > 90%). Similarly to previously shown results, dots represent the values of moisture content averaged from 25 repetitions and assigned to the specific time moment from the beginning of the moisture absorption/desorption experiment. Additionally, the black line reflects the fitted mathematical model and the grey contour symbolises the confidence region. Furthermore, where it was not possible to apply a mathematical model (R^2^ < 90%), boxplots revealing the spread of the recorded experimental data were used. Although information on the remaining peaks visible in the FT-IR spectrum has not been presented in the main part of the manuscript for the legibility reasons, data regarding all 16 absorption bands is available in Supplementary Material.

While analysing the data presented in Figure 5, it can be easily noticed that the greater number of linear models can be fitted for the peak no. 15 assigned to hydrophobic group, compared to the peaks no. 14 and 16, which are the signals originated from hydrophilic moieties. Moreover, models attributed to peak no. 15 exhibit higher adjusted determination coefficient values than for peaks no. 14 and 16, e.g., R^2^ determined for absorbance-based semilogarithmic model describing a moisture absorption experiment equalled approximately: peak no. 14%–94%, peak no. 15%–98%, peak no. 16%–96%. Similar trends could have been observed regarding the remaining mathematical regressions adjustments.

This observation could be explained with the nature of a certain peak. Often, maxima visible in IR spectrum are the common effect of many smaller signals that overlap. Therefore, their position and height are determined by many different interactions (Bledzki et al., 2002; Łojewska et al., 2005; Kondo et al., 2016), e.g., hydrogen bonds (Peršin et al., 2011), van der Waals forces (Missoum et al., 2013). However, peak no. 14 is assigned only to the moisture content in cellulose fibres. Hence, it reflects merely the vibrations of water molecules. Probably, this absorption band could be significantly affected by the cellulose-water interaction type (Park et al., 2006).

Moving forward, peak no. 16, exhibiting a better fit of different linear models, does not only simply reflect the interactions of hydroxyl moieties with water molecules. This broad absorption band could be successfully deconvoluted into three types of hydrogen bonding: intramolecular (3OH⋯O5 and 2OH⋯O6), intermolecular (6OH⋯O3′) (Oh et al., 2005a; Łojewska et al., 2005). Most likely, the position of this peak is stabilized with three species of interactions, two of which are related to the hydrogen bonds inside the cellulose macromolecule that are probably less affected by water presence. The synergistic effect of all interactions is the observable and describable change in the position of the peak over time. However, the regressions could have been only fitted in the experiment related to the moisture absorption process.

**FIGURE 6:**
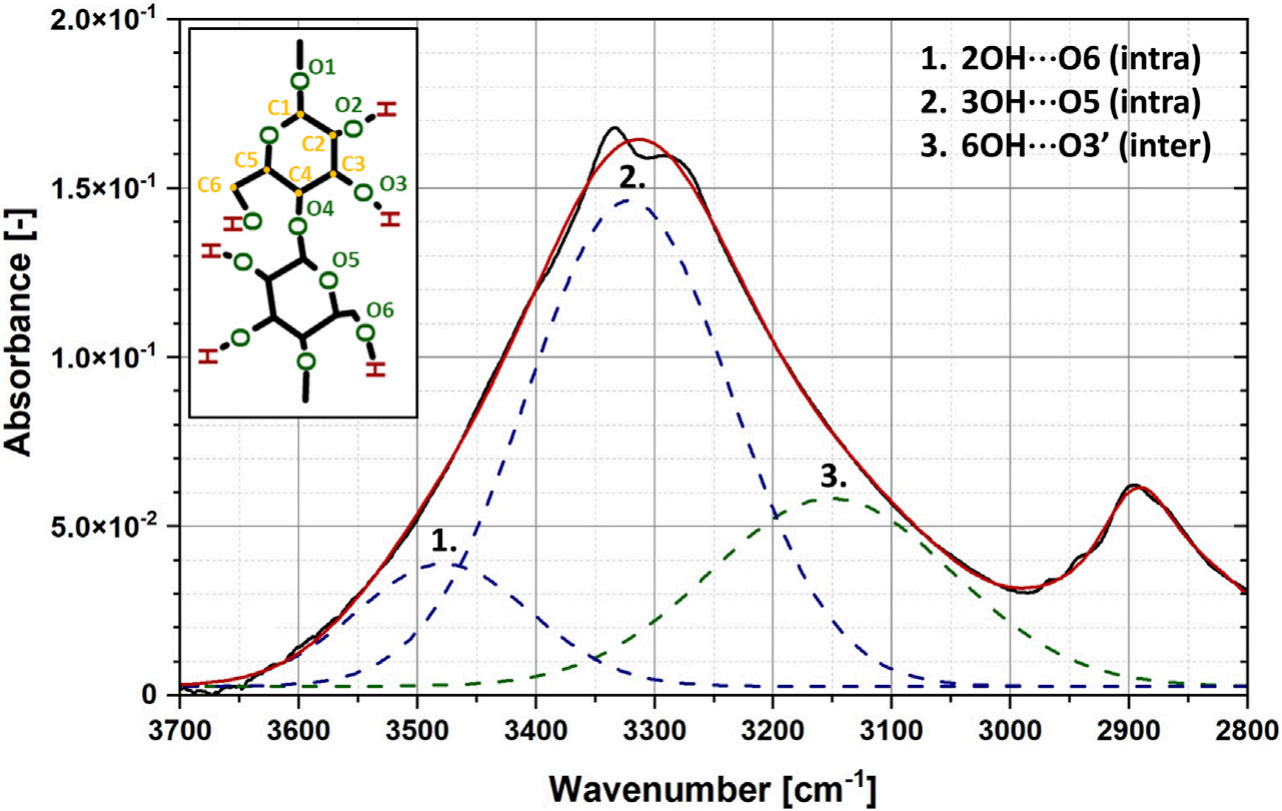
Exemplary deconvolution of the peak no. 16 assigned to hydroxyl moieties. Absorption band has been resolved into three types of hydrogen bonding: intramolecular (3OH⋯O5 and 2OH⋯O6), intermolecular (6OH⋯O3′).

In turn, peak no. 15 is very specific. As shown in Figure 6, it is located on the shoulder of a wider and larger absorption band (peak no. 16) assigned to the presence of hydroxyl groups (Oh et al., 2005a). Moreover, looking at the deconvolution of the peak no. 16 reported in the literature (Cichosz and Masek, 2020b) and presented in Figure 6, this shoulder is originated predominantly from 6OH⋯O3′ intermolecular hydrogen bond. This means one of the component signals of the peak no. 15 is the shoulder of the band that is likely to be significantly influenced by the presence of water molecules. Consequently, this specific position of the peak no. 15 may lead to its sensitivity to moisture content and the describable shifts along both *X* and *Y* axis. Contrary to peak no. 16, it is not disturbed by additional hydrogen interactions inside the cellulose macromolecule.

Based on the reasoning set out above, a certain conclusion can be drawn. When analysing the moisture content of cellulose fibres over time, the IR spectrum cannot be interpreted only in relation to bands assigned to specific chemical groups. The location of a given peak on the spectrum and its surroundings are very important. Therefore, in this study two peaks originated from non-polar and polar chemical moieties have been proposed for moisture content analysis based on the statistic-rooted analysis: peak no. 15 (2,898–2,888 cm^−1^) and peak no. 16 (3,339–3,327 cm^−1^).

### 3.5 Comparison of precision of the adopted analytical techniques in the measurement of cellulose water gain/loss and shortcomings in literature

Firstly, it should be noted that measurements of water content in natural fibres are not characterised by the highest possible repeatability (Gassan and Bledzki, 1997; Mihranyan et al., 2004a). Due to the fact that plant-based materials are synthesised by nature, they may differ in structure or contain defects. These, in turn, can affect the water content of cellulosic materials, as well as their hygroscopic capacity. Therefore, determining the precision of the measurements taken is crucial. Table 4 summarises selected research papers available in the literature, highlighting possible shortcomings in terms of the statistical operations used, which are relevant to the submitted dissertation.

**TABLE 4:** Comparison of applied statistics/mathematical operations in selected works dedicated to water content analysis in cellulose-based materials.

Subject of a study	Water content determination method	Applied statistics/mathematical operations	Shortcomings relevant to this article	Ref.
bacterial cellulose	gravimetric method	average (4 samples), standard deviation	a roughly defined value of the water content	Seifert et al. (2004)
wood	gravimetric method	average (13–38 samples), standard deviation	different number of samples for different tree species	Osunkoya et al. (2007)
	mobile nuclear magnetic resonance (NMR)	model calibration	not validated model; calibration error not presented; lacking: determination of a confidence area, *p*-value	Casieri et al. (2004)
	IR thermography (IRT)	calibration of the model for thermal effusivity dependence on water content	not validated model; calibration error not presented; lacking: determination of a confidence area, *p*-value	Ludwig et al. (2004)
	step-heating thermography, speckle patterns	model calibration	not averaged values (spread points presented in model calibration); not validated model; calibration error not presented; lacking: determination of a confidence area, *p*-value	Madruga et al. (2020)
	near infrared (NIR) spectroscopy, gravimetric methods	pre-processing of the spectra, model calibration (multivariate regression analysis) and validation	lacking: determination of a confidence area	Shukla and Sharma (2021)
plant cellulose	Karl-Fischer titration	none	only water content value has been given, no error calculation	Mazza et al. (2009)
cellulose acetate films	gravimetric method (water sorption isotherms)	model calibration	not validated model; calibration error not presented; lacking: determination of a confidence area, *p*-value	Chaurasia et al. (2010)
various plant fibres	attenuated total reflectance Fourier-transform infrared (ATR FT-IR) spectroscopy, gravimetric methods	pre-treatment of the spectra, model calibration and cross/external validation; provided: coefficient of determination, root mean square error	not averaged values (spread points presented in model calibration); lacking: determination of a confidence area, *p*-value	Célino et al. (2014)

The shortcomings reported in Table 4 do not imply a lack of reliability of the published articles. They merely indicate some scope for improvements in the presentation of results while enhancing the precision of the data presented. In the case of hardly reproducible measurements of water content in natural cellulose fibres (Gassan and Bledzki, 1997; Mihranyan et al., 2004a), it seems reasonable to indicate the confidence areas of the obtained results, coefficients of variation, and, for comparison purposes, to keep the same population of a given sample. In turn, when operating mathematical models, it is advisable to validate them.

Therefore, with a view to an accurate presentation of the results, an effort was made to eliminate the shortcomings shown in Table 4. This approach made it possible to compare the accuracy of the analytical methods used to analyse the water content of cellulose fibres in relation to the repeatability of the water absorption and desorption processes carried out. A comparison of the techniques analysed in this article can be found in the Table 5. It has been successfully proven that gravimetric methods allow for precise drawing of water absorption and desorption curves, while Karl-Fischer titration, ATR FT-IR and NIR techniques provide the possibility of the above processes’ description by linear mathematical models (R^2^ >90%). Moreover, presented data has been successfully supported by statistic-based approach enabling the assessment of investigated analytical techniques precision regarding the measurements with time.

**TABLE 5:** Summarised utility and the precision of the chosen analytical techniques in the analysis of cellulose moisture absorption and desorption processes.

Analytical technique	Intervals between the measurements	Plotting moisture absorption/desorption curve	Possibility of linear mathematical model adjusting	Precision of the technique^a^	
				Moisture absorption	Moisture desorption
Karl-Fischer titration	relatively short	yes; by intervals shortening	yes; only moisture absorption	higher at the beginning	higher at the end; increase from 1 h
gravimetric method	continuous measurement	yes	yes	higher at the end	higher at the end
thermogravimetric analysis (TGA)	long	yes; only for desorption curve (isothermal measurement)	no	coeff. of variation: 13.6%	coeff. of variation: 29.8%
attenuated total reflectance Fourier-transform infrared spectroscopy (ATR FT-IR)	short	questionable	yes	2,898–2,888 cm^−1^ (peak no. 15), 3,339–3,327 cm^−1^ (peak no. 16)	
near infrared spectroscopy (NIR)	short	questionable	yes	4,274–4,268 cm^−1^ (peak no. 1), 5,190–5,170 cm^-1^ (peak no. 4)	

^a^ Assessed based on boxplots presented.

## 4 Conclusion

The cellulose water absorption and desorption processes are characterised by low reproducibility (different structure of natural fibres, different availability of active centres, structural defects). Therefore, it is necessary to determine the precision of the analytical techniques used with respect to the poorly reproducible water absorption/desorption processes. Thus, in this work, the innovative statistic-based investigation of Karl-Fischer titration, (thermo)gravimetric analysis, attenuated total reflectance Fourier-transform infrared (ATR FT-IR) and near infrared (NIR) utility in cellulose moisture absorption/desorption processes analysis have been carried out. The gathered data was successfully described with boxplots revealing median, 1^st^ quartile and 3^rd^ quartile. Moreover, where applicable, linear mathematical models, as well as coefficients of variation were presented. On the basis of the collected results, it could have been clearly stated that different analytical techniques enable the description of the aforementioned processes with varied frequency and accuracy. Undoubtedly, the results presented in this study may contribute to the development of quantitative analysis of water content and tracking the processes of water absorption/desorption in cellulose-based materials. It was shown that gravimetric methods may contribute to drawing of water absorption and desorption curves, while Karl-Fischer titration, ATR FT-IR and NIR techniques provide the possibility of the above processes’ description by linear mathematical models (R^2^ >90%).

## Data Availability

The original contributions presented in the study are included in the article/Supplementary Material, further inquiries can be directed to the corresponding author.
