# Supplementary material for: Analysis of classical techniques precision on the measurement of cellulose moisture gain/loss
Source: Front Chem. 2023 Sep 6;11:1254941. doi: 10.3389/fchem.2023.1254941 (PMC10516550; doi:10.3389/fchem.2023.1254941)
Supplement: Supplementary file 1 [file DataSheet1.PDF]

Supplementary Material

Analysis of classical techniques precision on the measurement of cellulose moisture gain/loss

Stefan Cichosz[1], Katarzyna Dems-Rudnicka[2], Anna Masek[1*]

[1] Lodz University of Technology, Institute of Polymer and Dye Technology, Faculty of Chemistry, 90-537 Lodz, Stefanowskiego 16, Poland

[2] Lodz University of Technology, Centre of Mathematics and Physics, 90-924 Lodz, Politechniki 11, Poland

*** Correspondence:**
Anna Masek
anna.masek@p.lodz.pl

1 Additional results

1.1 Fourier-transform infrared spectroscopy (FT-IR)

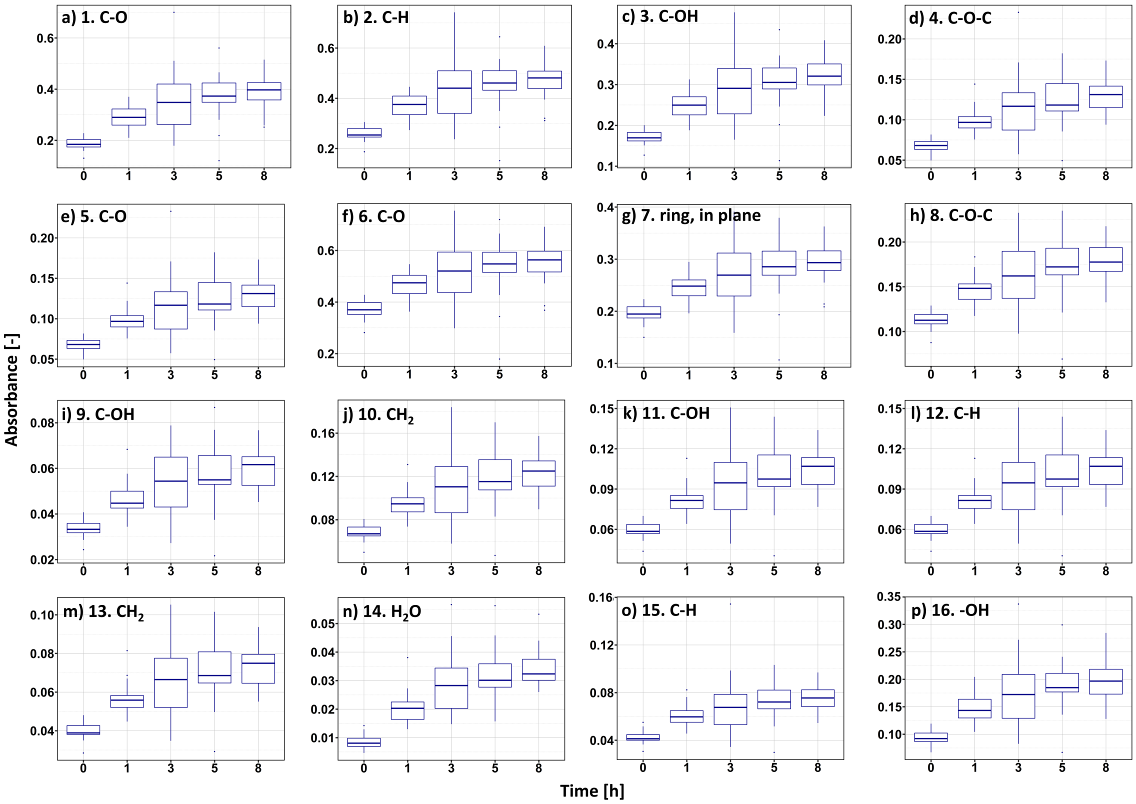

Fig. S1 Height of the selected peaks (a-p) visible in ATR FT-IR spectrum plotted as a function of the moisture absorption time.

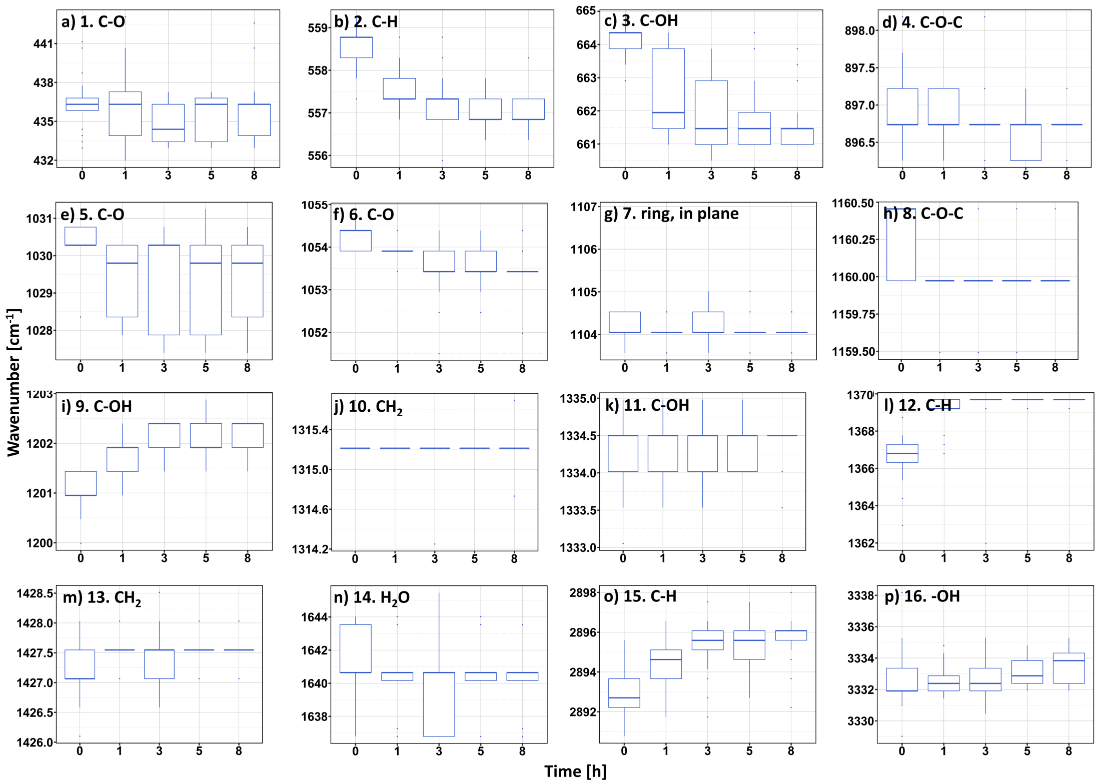

Fig. S2 Wavenumber of the selected peaks (a-p) visible in ATR FT-IR spectrum plotted as a function of the moisture absorption time.

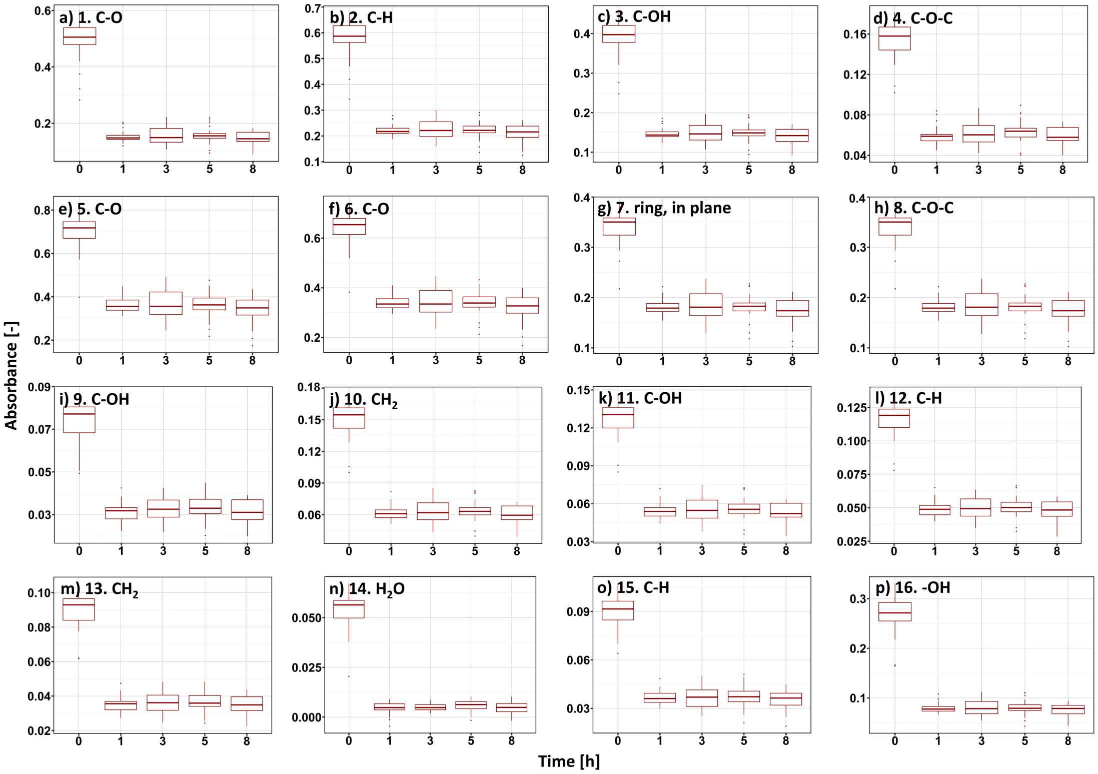

Fig. S3 Height of the selected peaks (a-p) visible in ATR FT-IR spectrum plotted as a function of the moisture desorption time.

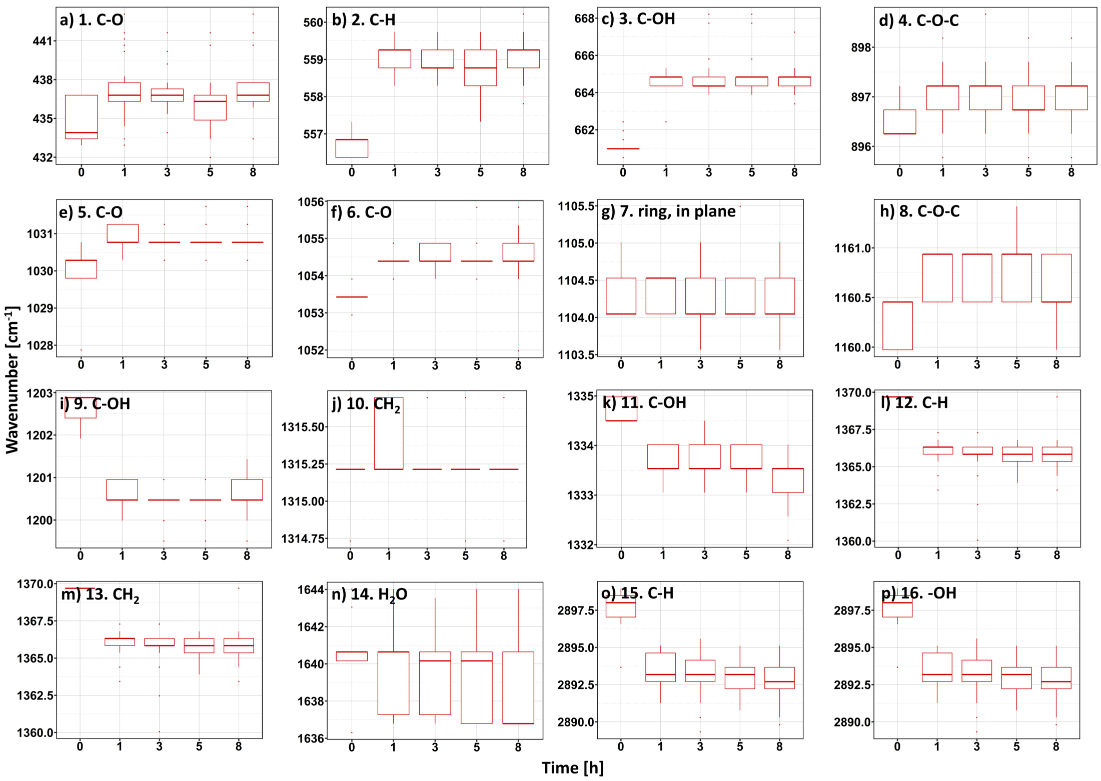

Fig. S4 Wavenumber of the selected peaks (a-p) visible in ATR FT-IR spectrum plotted as a function of the moisture desorption time.

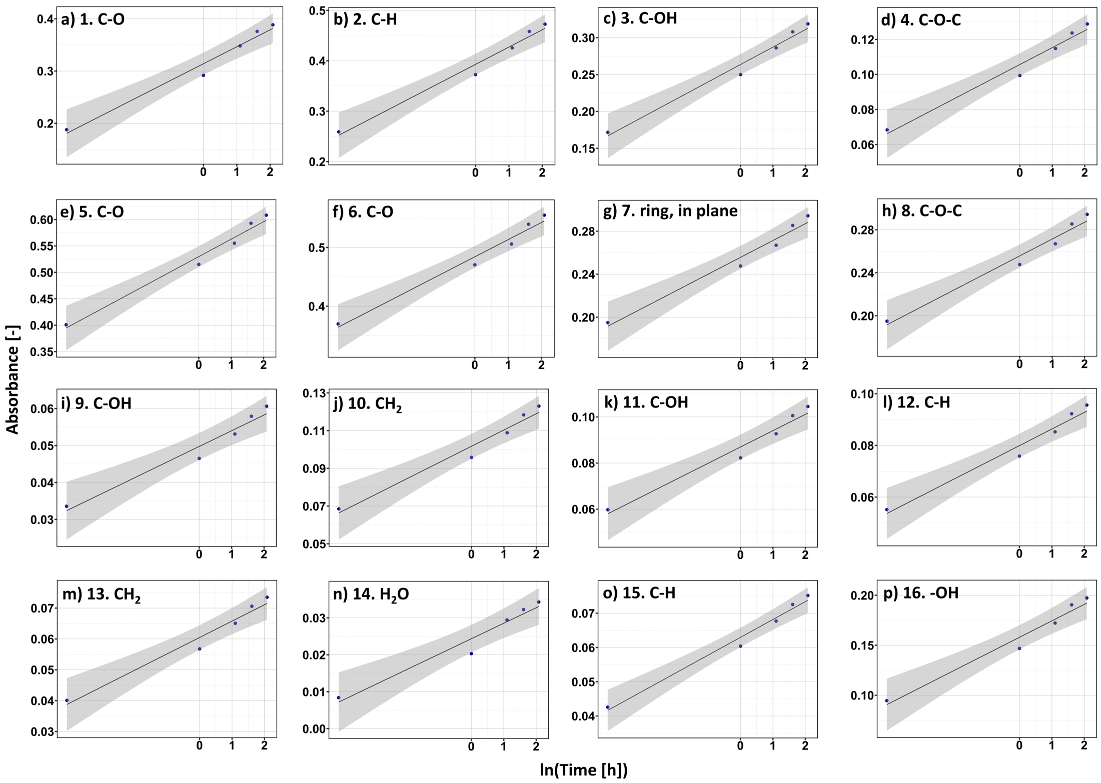

Fig. S5 Possible models describing absorbance-ln(time) relationship for the selected peaks (a-p) visible in ATR FT-IR spectra recorded during moisture absorption process.

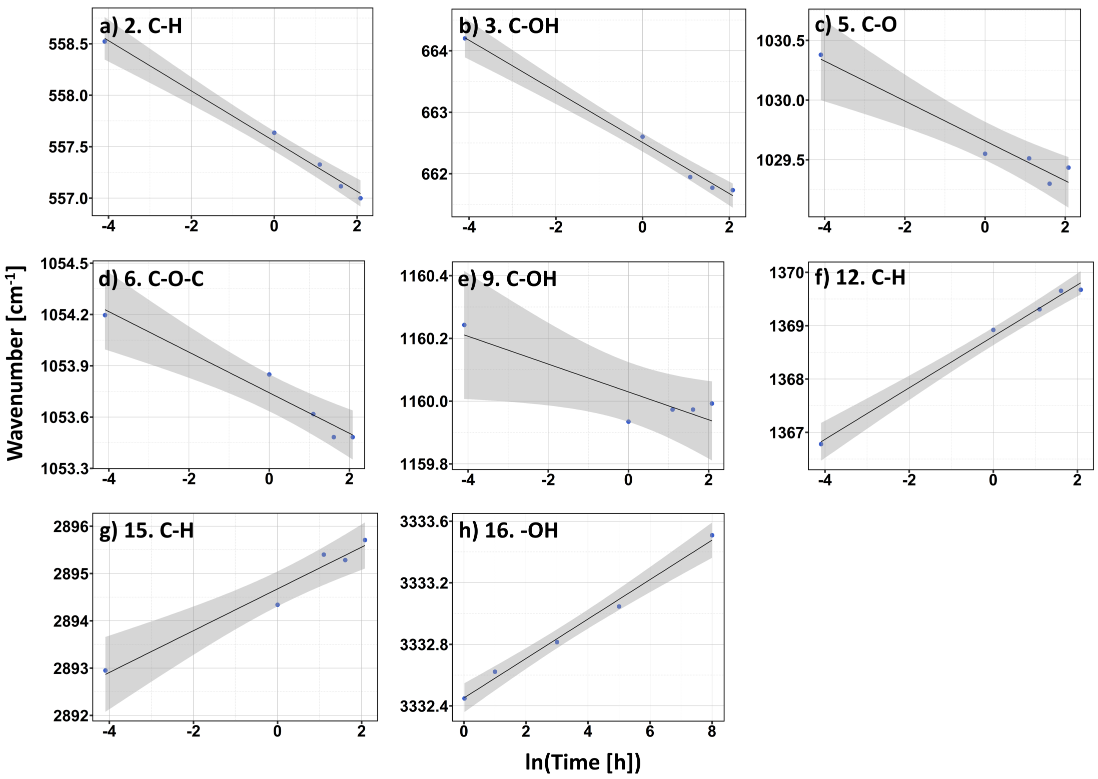

Fig. S6 Possible models describing wavenumber-ln(time) relationship for the selected peaks (a-h) visible in ATR FT-IR spectra recorded during moisture absorption process.

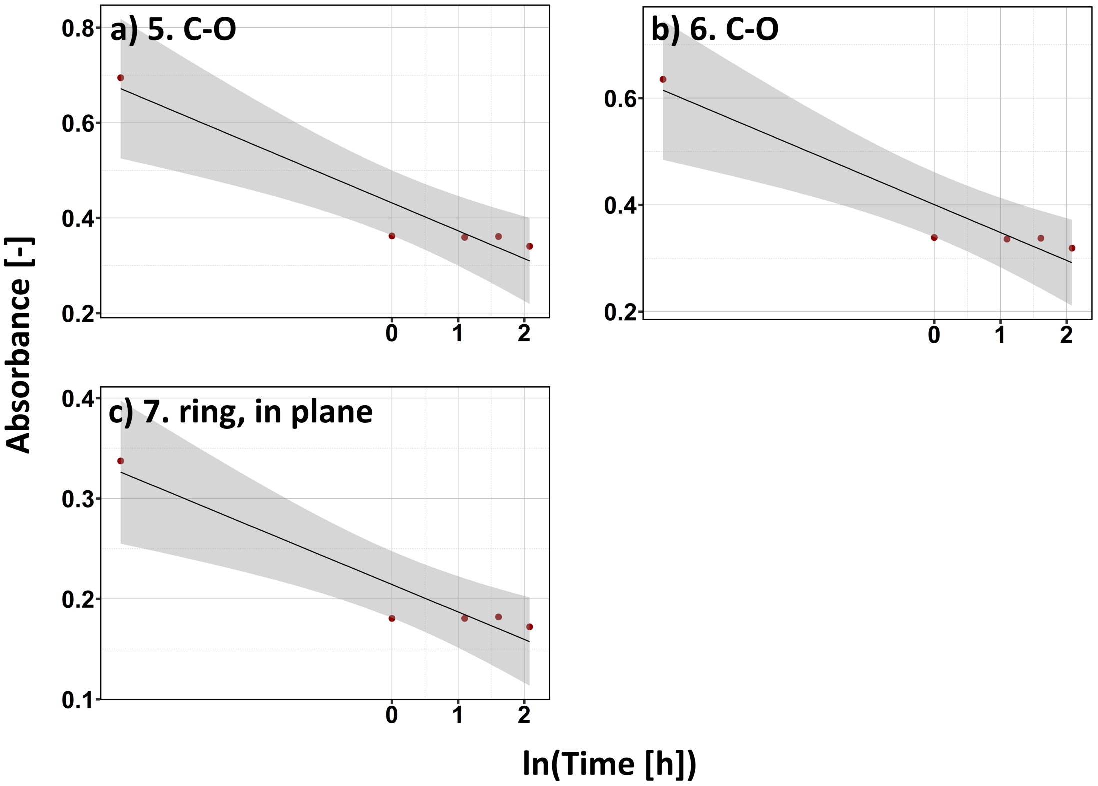

Fig. S7 Possible models describing absorbance-ln(time) relationship for the selected peaks (a-c) visible in ATR FT-IR spectra recorded during moisture desorption process.

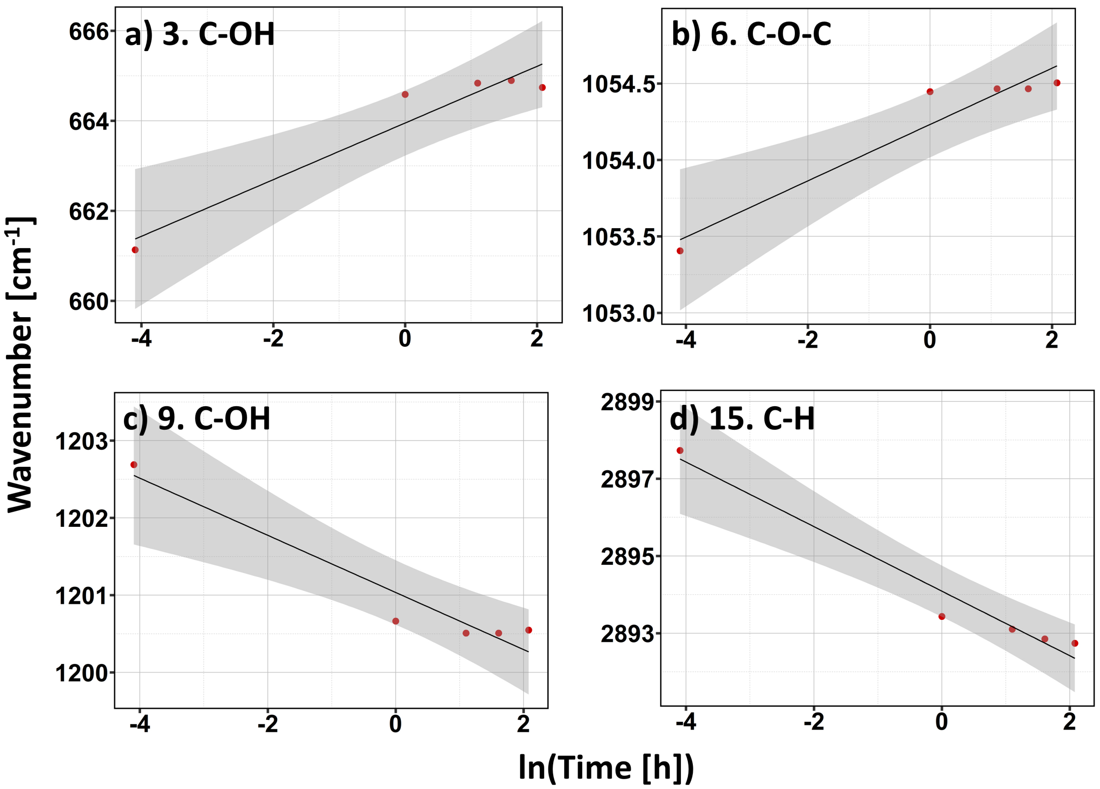

Fig. S8 Possible models describing wavenumber-ln(time) relationship for the selected peaks (a-d) visible in ATR FT-IR spectra recorded during moisture desorption process.

1.2 Near infrared spectroscopy (NIR)

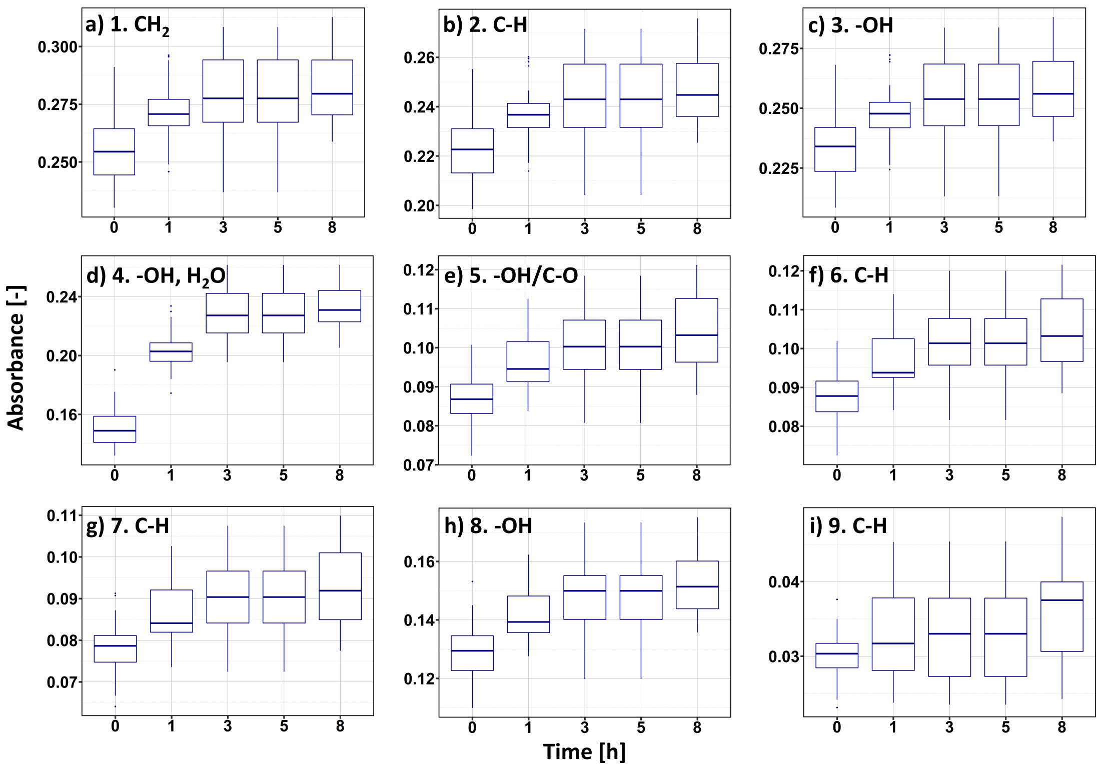

Fig. S9 Height of the selected peaks (a-i) visible in NIR spectrum plotted as a function of the moisture absorption time.

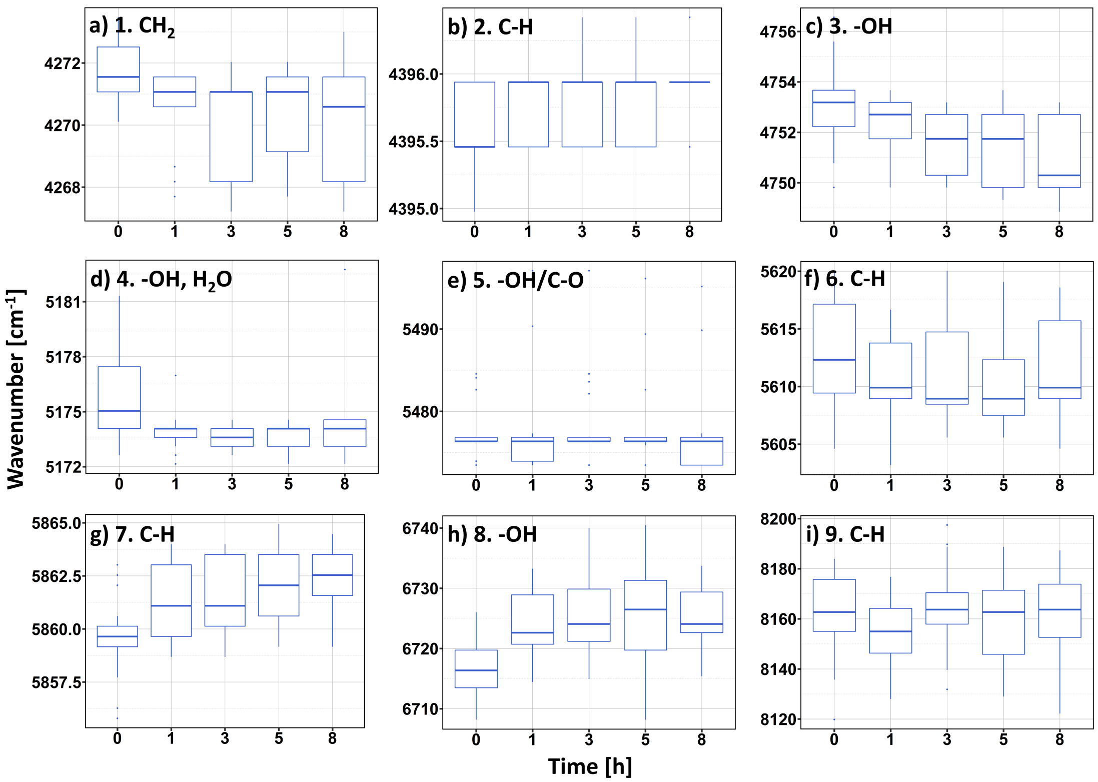

Fig. S10 Wavenumber of the selected peaks (a-i) visible in NIR spectrum plotted as a function of the moisture absorption time.

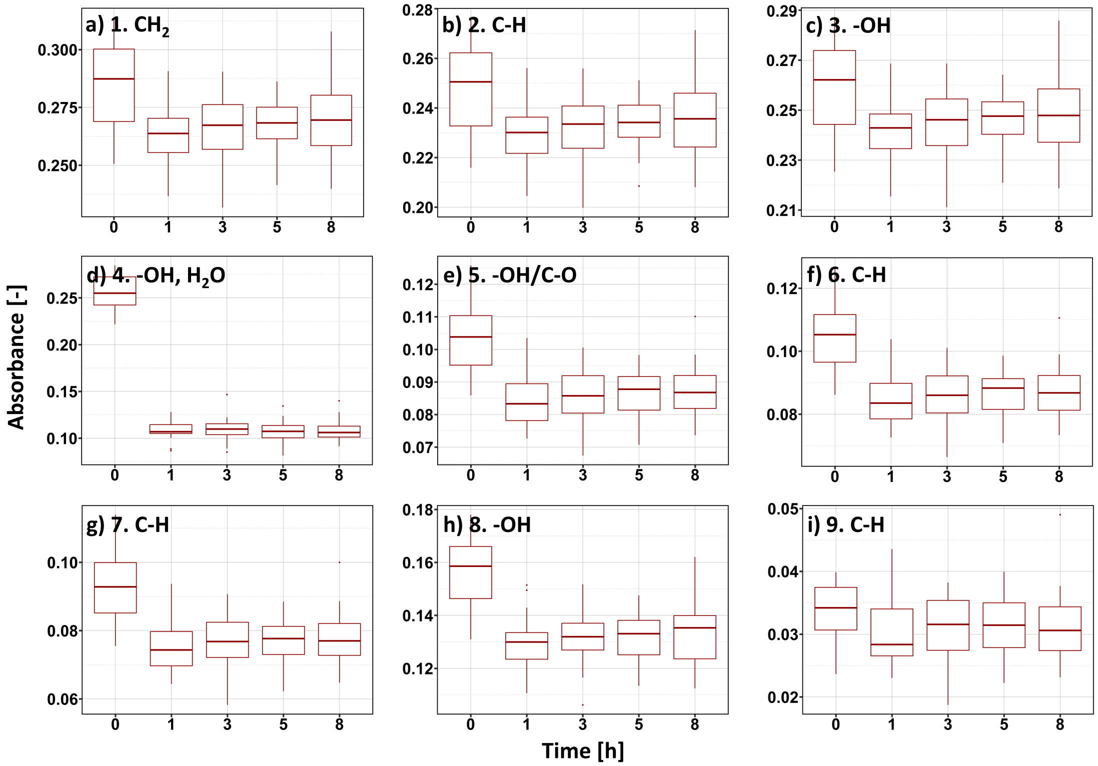

Fig. S11 Height of the selected peaks (a-i) visible in NIR spectrum plotted as a function of the moisture desorption time.

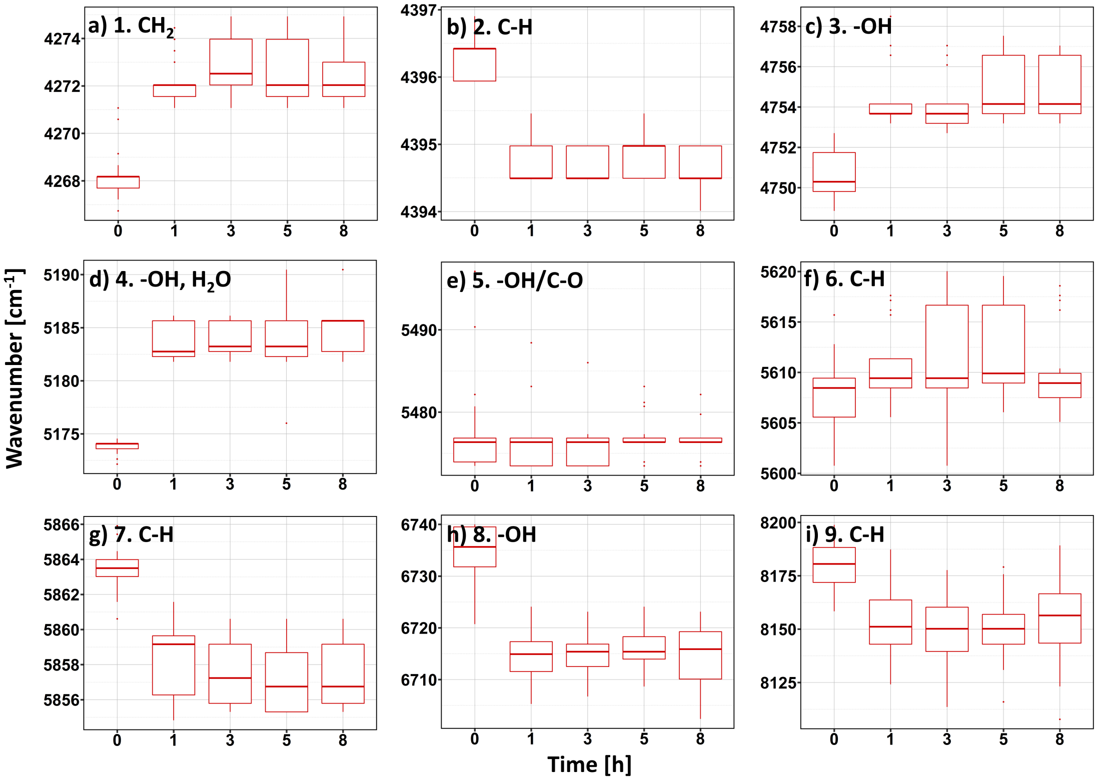

Fig. S12 Height of the selected peaks (a-i) visible in NIR spectrum plotted as a function of the moisture desorption time.

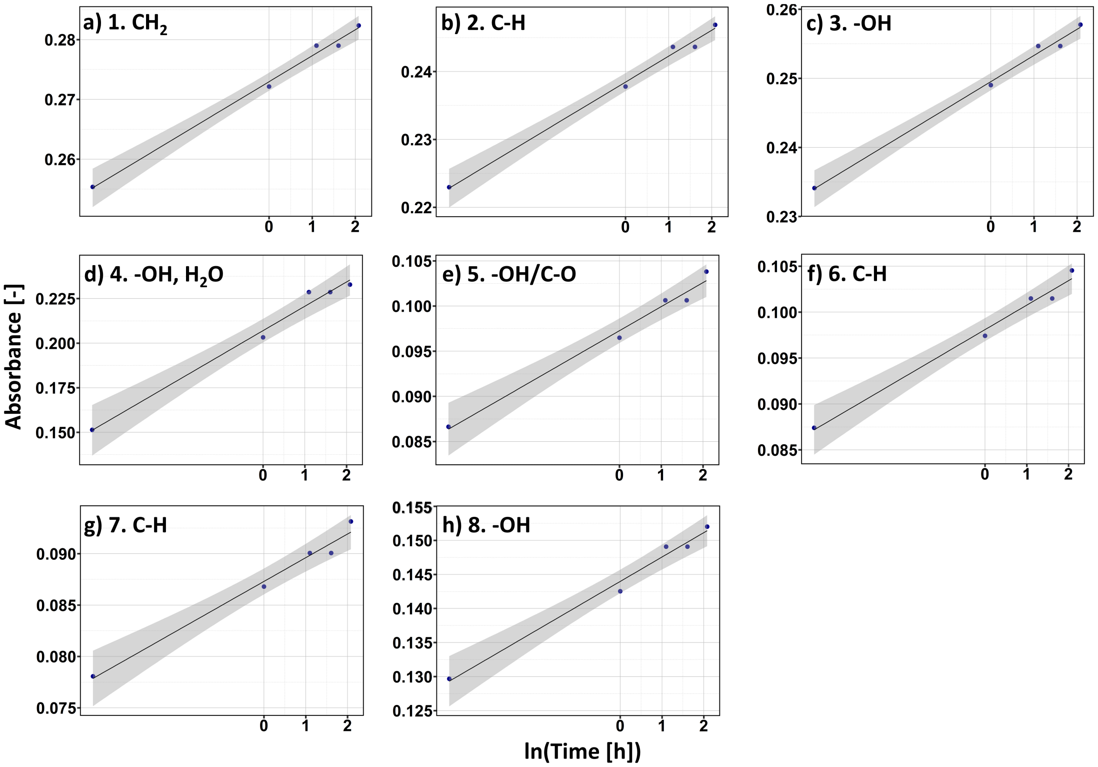

Fig. S13 Possible models describing absorbance-ln(time) relationship for the selected peaks (a-h) visible in NIR spectra recorded during moisture absorption process.

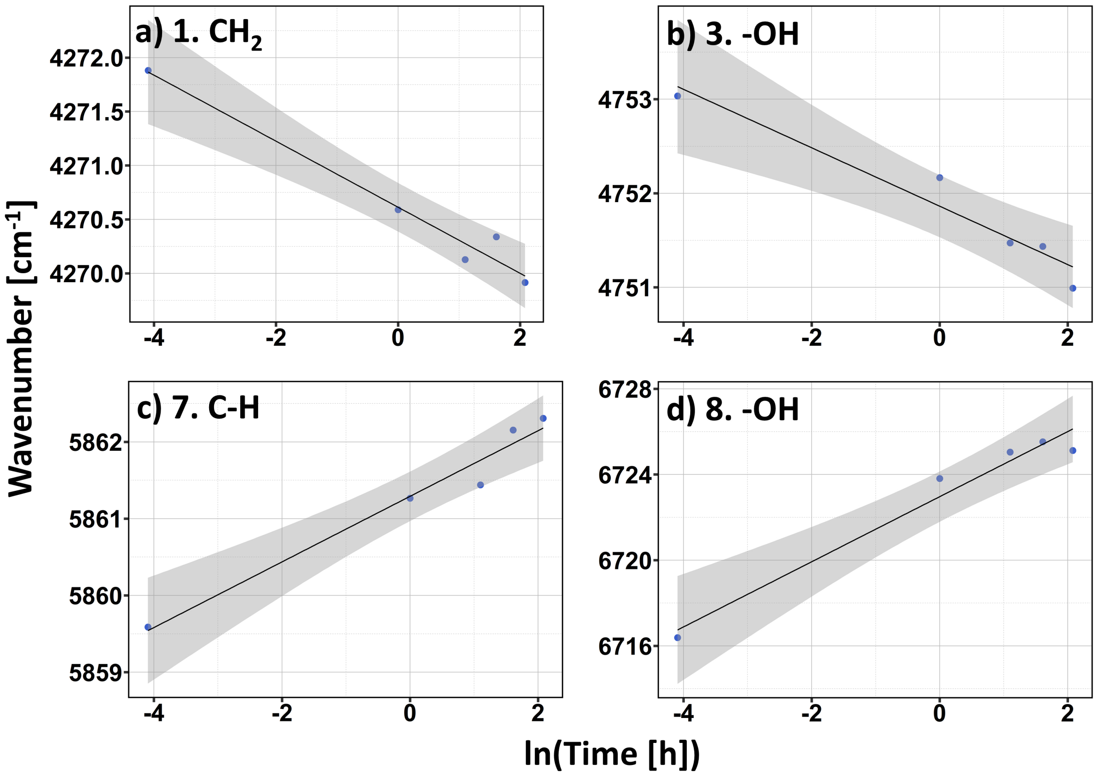

Fig. S14 Possible models describing wavenumber-ln(time) relationship for the selected peaks (a-d) visible in NIR spectra recorded during moisture absorption process.

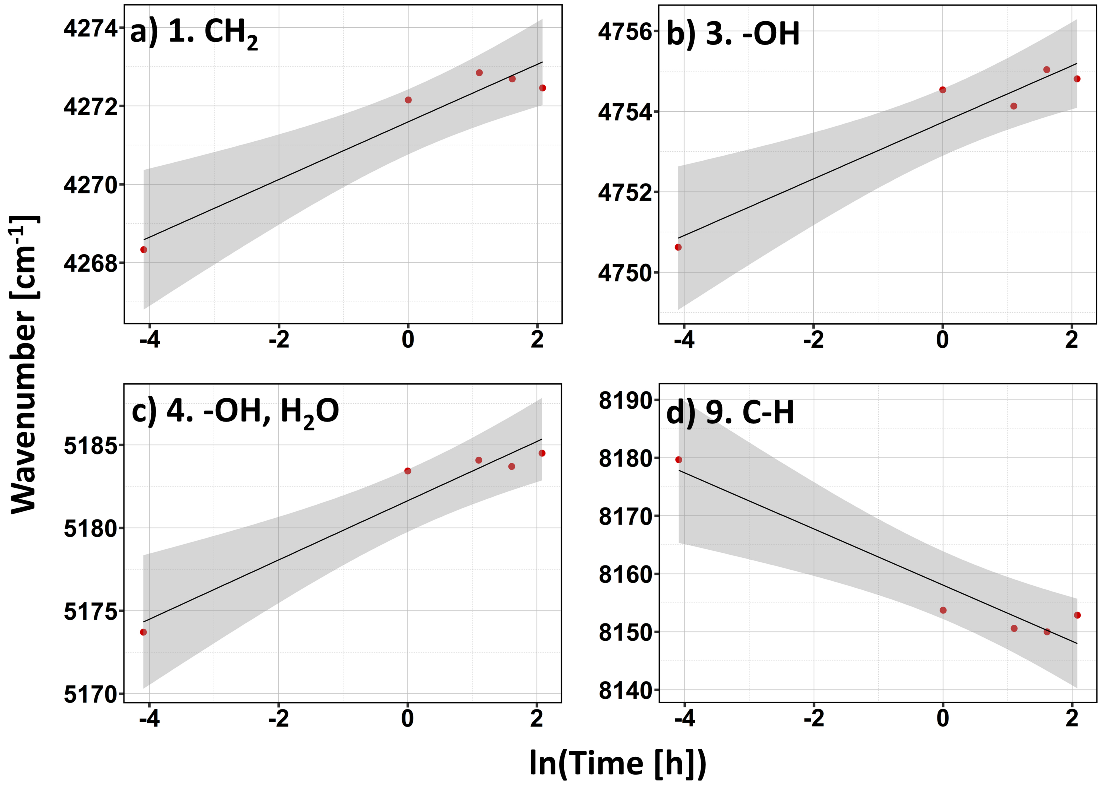

Fig. S15 Possible models describing wavenumber-ln(time) relationship for the selected peaks (a-d) visible in NIR spectra recorded during moisture desorption process.
